# Ascidian Toxins with Potential for Drug Development

**DOI:** 10.3390/md16050162

**Published:** 2018-05-13

**Authors:** Dianne J. Watters

**Affiliations:** School of Environment and Science, Griffith University, Brisbane, Queensland 4111, Australia; d.watters@griffith.edu.au; Tel.: +61-7-3378-8895

**Keywords:** secondary metabolites, bacterial symbiosis, anticancer activity, mechanism of action

## Abstract

Ascidians (tunicates) are invertebrate chordates, and prolific producers of a wide variety of biologically active secondary metabolites from cyclic peptides to aromatic alkaloids. Several of these compounds have properties which make them candidates for potential new drugs to treat diseases such as cancer. Many of these natural products are not produced by the ascidians themselves, rather by their associated symbionts. This review will focus mainly on the mechanism of action of important classes of cytotoxic molecules isolated from ascidians. These toxins affect DNA transcription, protein translation, drug efflux pumps, signaling pathways and the cytoskeleton. Two ascidian compounds have already found applications in the treatment of cancer and others are being investigated for their potential in cancer, neurodegenerative and other diseases.

## 1. Introduction

Ascidians (urochordates, tunicates), commonly known as sea-squirts, belong to the Phylum, Chordata; sub-phylum; Tunicata; class, Ascidiacea. The chordates consist of three lineages, the urochordates, the cephalochordates, and the vertebrates. Ascidians are benthic filter-feeding protochordates ancestral to the higher chordates. The swimming larval stages contain a notochord, however the adults are invertebrates and are immotile. This section will provide an overview of these organisms and the natural products isolated from them, as well as outline the scope of this review.

### 1.1. Overview of Ascidians

Ascidians are exclusively marine, abundant in harbors, and can be found all over the world from near the surface to great depths [1,2,3]. The three orders within the class Ascidiacea, based on the structure of the adult branchial sac, are Aplousobranchia (almost exclusively colonial), Phlebobranchia, and Stolidobranchia (in both solitary and colonial forms) [4]. Several species of Stolidobranchs are farmed for food in some parts of the world, mainly Japan and Korea [2] and *Pyura stolinifera* (commonly called cunjevoi) is widely used as fishing bait in Australia. There are over 3000 species of ascidians [4] and they exist below low-tide levels in protected areas with good water movement. Many can tolerate and accumulate heavy metals, although these metals affect the development of embryos and larvae in a dose-dependent fashion. This makes certain species of ascidians useful as indicators of water quality in bioassays for pollutants [5]. In addition, ascidian embryos are also useful as a model to study the neurodevelopmental toxicity of different compounds [6]. Several families of ascidians accumulate very high levels of vanadium (up to 350 mM) in tissues and blood cells called vanadocytes. The physiological function of the accumulated vanadium is presently unclear. It has been suggested that it may be involved in chemical defense against predators and microbes, or in metabolic roles such as oxidation and reduction reactions [7]. Ascidians are important ecologically due to their invasive potential and adverse effects on native fauna and aquaculture [2,3]. The ecological role of ascidians and the potential of using invasive species for marine natural product discovery and production has been reviewed [8]. LC-MS metabolomics has been used recently to identify 71 metabolites in the invasive ascidian *Styela plicata*. Fractions were assayed for antitumor and apoptosis inducing properties, revealing many molecules with potential awaiting further research [9].

### 1.2. Ascidian Natural Products

Ascidians, along with sponges and bryozoans, produce a rich variety of secondary metabolites presumably to avoid predation and as an anti-fouling mechanism. These include cyclic peptides and depsipeptides and many different types of aromatic alkaloids. Many of these metabolites are not produced by the ascidian themselves but by endosymbiotic micro-organisms.

In recent years, a considerable number of reviews on the diverse natural products isolated from marine invertebrates, including ascidians, have been published. There are several reviews on marine peptides covering the structural diversity and clinical applications of marine cyclic peptides [10], marine peptides as anticancer agents [11,12], proline-rich cyclic peptides [13], and marine peptides with therapeutic potential [14]. Anti-tumor compounds from marine sources with information on their mechanism of action (apoptosis, anti-angiogenesis, microtubules, anti-proliferative) are discussed by Ruiz-Torres and colleagues [15]. Further reviews covered the biosynthesis and biological activities of marine pyridoacridine alkaloids [16] and alkaloids from marine sources as anticancer agents [17]. Several reviews deal specifically with ascidian natural products: Palanisamy and coworkers have provided a comprehensive treatise on approximately 580 ascidian compounds isolated from 1994 to 2014 [18], dealing with their structure and reported biological activity (antibacterial, anti-inflammatory, anti-viral, anti-diabetic, anti-proliferative, anti-parasitic). In addition, there are reviews on bioactive peptides from ascidians [19], ascidians as a source of anticancer agents [20], and the pharmacological potential of non-ribosomal peptides from ascidians and sponges [21].

The ascidian families Didemnidae, Polyclinidae, and Polycitoridae are the most prolific producers of bioactive compounds with diverse activities and potential for development as therapeutic drugs for a wide variety of diseases [18]. There has already been some notable success with two ascidian natural products marketed for cancer treatment. Ecteinascidin (ET-743, trabectedin) from *Ecteinascidia turbinata* is FDA approved and marketed under the trade name Yondelis^®^. Aplidin^®^ (dehyrodidemnin B, plitidepsin)—first isolated from *Aplidium albicans*—has attained orphan drug status [22]. Both are marketed by PharmaMar (Madrid, Spain).

### 1.3. Purpose of This Review

For the majority of compounds, only a simple cytotoxicity assay in a panel of human cancer cell lines is reported, with little or no information on their mode of action. In the present review therefore, the focus will be on ascidian toxins of pharmacological interest, with an emphasis on the mechanism of action of important ascidian compounds, concentrating mainly on the literature since 2014 up to February 2018. The current review will cover recent advances in our understanding of ascidian-associated symbionts, biosynthesis, and mechanism of action of a variety of ascidian natural products including cyclic peptides and depsipeptides, polyketides, and diverse alkaloids. Toxins affecting signal transduction pathways and the cytoskeleton will also be discussed. In addition, reference will be made to newly isolated compounds and to advances in the chemical synthesis of the natural products and their analogues for structure–activity relationships and drug development.

## 2. Symbiotic Organisms in the Biosynthesis of Ascidian Natural Products

Symbiotic bacteria contribute secondary metabolites necessary for defense and the survival of ascidians [23,24]. About 80 of the currently known secondary metabolites from ascidians are made by symbiotic bacteria [25]. These metabolites are essential for the interaction between host and symbiont, and the bacteria are phylogenetically diverse [26].

### 2.1. Microbial Diversity

The discovery processes for ascidian microbial symbionts range from the traditional and culture-dependent to the metagenomic and next generation sequencing approaches [25]. Next generation sequencing has provided comprehensive information about ascidian microbial diversity. Microbes associated with ascidians are species specific, and tissue specific, for example gut vs. tunic [25]. Bacterial and chemical analyses of 32 different didemnid ascidians was performed, comparing the metabolomes and microbiomes across geographical locations. It was found that ascidian microbiomes are highly diverse with the diversity not correlated to geographical location or latitude [27]. The microbiome of introduced ascidians was also found to be species specific and likely contributes to the host’s adaptation to the new environment [28]. A recent study investigated the *Actinomycetes* species associated with three Australian ascidians [29]. Species specificity was again observed and mass spectrometry analysis revealed that many of the metabolites were likely to be synthesized by the *Actinomycetes*. The highly diverse metabolome of the *Actinomycetes*, particularly the *Streptomyces* and *Micromonospora*, may provide a rich source for further natural product discovery from the cultured organisms. The bacterial producers of most ascidian compounds remain unknown. However, where the producer is known, it usually is one of the top ten strains in the microbiome [27].

### 2.2. Prochloron

In the family Didemnidae, cyanobacteria *Prochloron didemni* produce a variety of toxic cyclic peptides known as cyanobactins, over 60 in number [30,31]. *Lissoclinum patella* hosts two cyanobacteria *Prochloron,* and *Acaryochloris* [32]. *Prochloron* are obligate symbionts and cannot survive outside the host, hence attempts to culture them have been unsuccessful. There are about 30 species of host ascidians all belonging to four genera of the family *Didemnidae* (*Didemnum, Trididemnum, Lissoclinum*, and *Diplosoma*) [33,34]. Hirose reviewed the diversity of modes of symbiont transmission across generations in the *Prochloron* ascidian photosymbionts [34]. The cyanobacterial symbionts are actively exchanged (horizontal transmission) among the host colonies, resulting in a high level of symbiont genetic diversity in a single host colony.

## 3. Cyanobactins

Cyanobactin was the name given to a diverse group of cyclic peptides of 6–8 amino acids, which contain heterocyclized amino acids (oxazolines, thiazolines, or their oxidized derivatives oxazoles and thiazoles) and/or isoprenoid amino acid derivatives [30]. Cyclic peptides which consist solely of amino acids used in protein synthesis are also included [31]. Table 1 in the article by Sivonen et al. [31] provides a list of the cyanobactins.

### 3.1. Biosynthesis of Cyanobactins

**Figure 1:**
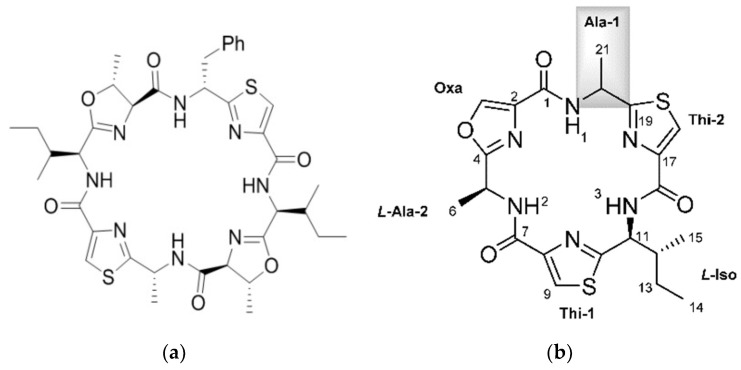

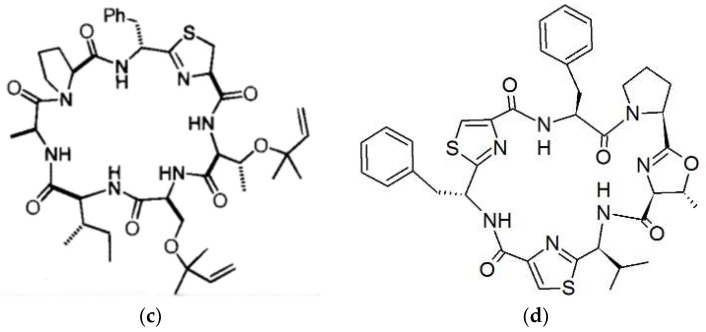
Selected Cyanobactins from Didemnid Ascidians. (**a**) patellamide D, reproduced from [49]; (**b**) bistratamides M and N; reproduced from [45]; (**c**) trunkamide A; adapted with permission from (Wipf, P.; Uto, Y. *J. Org. Chem*. 2000, 65, 1037, [50]) Copyright 2018 American Chemical Society (Washington, DC, USA); (**d**) Lissoclinamide 5.

Cyanobactins are made on the ribosome by the RiPP mechanism (ribosomally synthesised and post-translationally modified peptides). For example, the patellamides and trunkamides [35,36]. A selection of cyanobactins is illustrated in Figure 1. The biosynthetic enzymes for cyclic peptide synthesis are encoded in the *Prochloron* genome [36]. Precursor peptides are post-translationally modified by enzymes adding heterocycles derived from Cysteine, Serine, and Threonine and/or isoprene units [37]. The modified peptides are then cleaved from the precursor and cyclized to the natural products. These natural products often exhibit combinatorial biosynthesis [37]. RiPP combinatorial chemistry is made possible due to core peptide hypervariability, broad substrate specificity, enzyme recognition sequences, and modularity of post-translational elements. Many novel post translational modifications are also found in marine organisms [38]. The mechanisms and gene clusters involved in the formation of the thiazoline and oxazoline rings in the cyanobactins have been well studied [39]. The patellamide pathway, coded by the *pat* gene cluster—which is constitutively expressed in *Prochloron*—involves several enzymatic steps: amino acid heterocyclization, peptide cleavage, peptide macrocyclization, heterocycle oxidation, and epimerization. Some closely related products are also prenylated. The enzyme activities have been identified for all these transformations except epimerization, which may be spontaneous [40]. An additional pathway to the patellamide pathway is the trunkamide (*tru*) pathway [35]. Two new pathways have been identified by Lin and coworkers—the pat-like *bis* cluster for bistratamides and the *tru*-like, *trf* cluster encoding patellins [41]. Using whole-genome data it was shown that there is a close and specific relationship between the *Prochloron* symbiont and the host as they have congruent phylogenies. There was no relationship between *Prochloron* and environmental habitat, as animals from the same habitat had different *Prochloron* strains. This important work also showed how these pathways could generate diversity of cyanobactins by swapping core peptides and enzymes, which have broad substrate tolerance.

Each enzyme in the *pat*-like and *tru*-like pathways has been crystallized and analyzed. Expression of the cyanobactin trunkamide pathway was recently achieved using rational engineering and empirical methods [36,42]. To show how chemical diversity can be generated from a metabolic pathway, a model has been developed using the *tru* pathway. Each metabolic step is slower than in conventional pathways and intermediates are long lived, accumulating progressively. With broad substrate tolerance, these diversity-generating pathways may allow the organism to adapt to changing predators by altering toxic metabolites [42]. The discovery of these pathways opens the way for large scale production of cyanobactins by protein engineering and in vitro [43]. New methods for accurate quantitation of synthesized cyanobactins have been developed without the need for authentic standards [43].

### 3.2. Biological Activity of Cyanobactins

In the comprehensive review of marine peptides by Gogenini and Hamann, a list of the cyclic peptides isolated from ascidians along with their biological activity and IC_50_ values is provided [14]. For the cyanobactins, this is typically in the micromolar range. The role of the cyanobactins is currently unknown. Some are cytotoxic to mammalian cells in culture and some bind metals such as Cu(II) and Zn(II), however the role of the metal complexes is not clear. The copper concentration of ascidians is several orders of magnitude higher than seawater leading to the idea that some cyanobactins function in copper transport and storage, or detoxification [44]. Two novel cyclic hexapeptides—bistratamides M and N—differing only in the configuration of one alanine side chain (Figure 1) have been isolated [45]. In the same study the authors also examined the metal binding of Bistratamide K and showed that it binds Zn(II). Patellamides can bind two copper atoms. The structure of copper carbonate complexes of patellamides has been determined, suggesting a role in CO_2_ hydration (carbonic anhydrase reactivity) providing carbonate for attachment or CO_2_ for photosynthesis [46,47]. One of the complexes catalyzed hydration of CO_2_ at a rate only two orders of magnitude lower than the enzyme carbonic anhydrase. Other proposed roles include oxygen activation and phosphoester hydrolysis (phosphatase reactivity), and acting as enzyme co-factors [35]. Using a patellamide derivative with an appended fluorescence tag, it was found that Cu is coordinated to the patellamides inside the *Prochloron* cells [48].

A new study examined the metal binding of synthetic heteroatom-interchanged (HI)-lissoclinamide 5, whereby the carboxamide group at position 4 on the 1,3-thiazole ring system of lissoclinamide 5 was moved to position 5, producing a nitrogen/sulfur heteroatom interchange so that the sulfur atoms pointed to the center of the cavity [51]. The HI cyclic peptide showed poor copper binding affinity with the S donor of the thiazole not involved in coordination. There was also lower cytotoxicity (by an order of magnitude) compared to the parent compound. This study also identified the most likely structure for the Cu(II) complex with natural lissoclinamide 5, in which the metal ion is bound through the nitrogen donors of the two thiazoles and a deprotonated amide.

Two new dimeric hexapeptides—antollamides A and B—have recently been isolated from the ascidian *Didemnum molle* [52]. These are the only cyanobactins that have intermolecular dimerization through disulfide bonds. However, they lack significant cytotoxicity.

## 4. Cyclic Depsipeptides and Polyketides

In cyclic depsipeptides, the ring is mainly composed of amino- and hydroxy acid residues connected by amide and ester bonds (at least one of the latter) [53]. These compounds and some alkaloids are made by the non-ribosomal peptide synthesis (NRPS) mechanism. Polyketides are complex molecules built from simple carboxylic acids and synthesized by polyketide synthetases (PKS). NRP and polyketide synthetases are large multienzyme machineries which have been reviewed recently [54,55]. Natural products having polyketide and non-ribosomal peptide structures are generally found to be of microbial origin.

### 4.1. Didemnins

Didemnins are cyclic depsipeptides with highly modified amino acid residues. Didemnin B (first isolated from *Tridemnum solidum* [56]) has previously been shown to bind the GTP-bound form of eukaryotic elongation factor 1A (eEF1A), inhibiting its release from the ribosomal A site and preventing peptide elongation [57]. Several additional actions of this compound have been identified. For example, it activates the mammalian target of rapamycin (mTORC1) pathway through release of REDD1 inhibition. REDD1 (Regulated in development and DNA damage response 1) is a short-lived protein and its levels decline due to inhibition of protein synthesis. Didemnin B also inhibits palmitoyl-protein thioesterase (PPT1) and the dual inhibition of PPT1 and eEF1A results in induction of apoptosis through loss of the protective Mcl-1 protein [58]. Didemnin B is also a powerful immunosuppressant, 100 times more potent than cyclosporine A [59]. Numerous clinical trials were conducted with didemnin B. However, these were discontinued due to significant toxicity.

**Figure 2:**
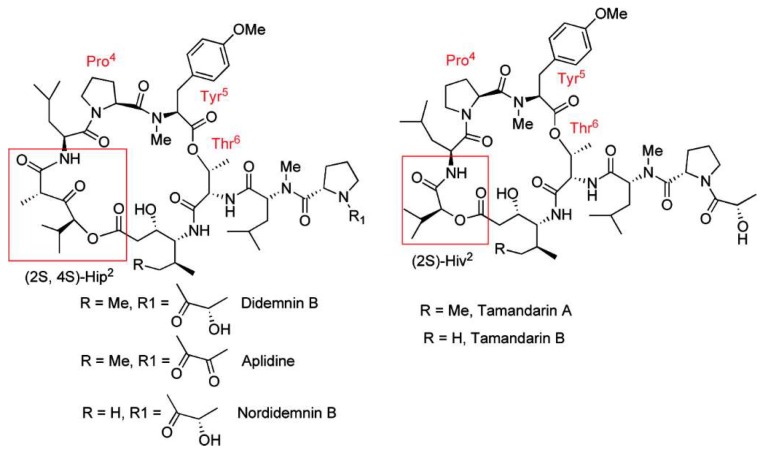
Structures of didemnins and tamandarins. The tamandarins differ from the didemnins by replacement of the Hip (α-(α-hydroxyvaleryl)-proprionyl) moiety with Hiv (α-hydroxyvaleryl). Reproduced with permission from (Adrio, J. et al., *J. Org. Chem*. 2007, 72, 5129. [71]) Copyright 2018 American Chemical Society (Washington, DC, USA).

Plitidepsin, (dehydrodidemnin B) is an analog in which a lactyl group of didemnin B is replaced by a pyruvyl group and was first isolated from *Aplidium albicans* as shown in Figure 2. It is marketed under the name Aplidin^®^ and is in advanced clinical trials for several malignancies such as multiple myeloma [60]. Trials of Aplidin^®^, with and without dexamethasone, have been completed and another in combination with both dexamethasone and bortezomid is currently recruiting participants [61]. Aplidin^®^ received orphan drug status for treating multiple myeloma in May 2017 and PharmaMar has requested re-examination of its use for relapsed and refractory multiple myeloma by the European Medicines Agency in January 2018 [62].

The primary target of plitidepsin (Aplidin^®^) is thought to be eukaryotic elongation factor 1A2 and this factor is commonly depleted in plitidepsin resistant cells [60,63]. The drug targets the non-canonical roles of eEF1A2 [63]. Binding of plitidepsin to eEFA2 occurs when the protein is in the GTP-bound conformation. It also binds eEF1A but with a lower K_d_ (180 nM for A1 vs. 80 nM for A2) and the interaction has been observed in living cells using a FLIM-phasor FRET approach [63]. The events which trigger cell death, and how they are linked with eEF2A binding, are yet to be elucidated. Plitidepsin was shown to cause cell cycle arrest and induce apoptosis in melanoma cells through activation of Rac1/JNK [64]. Apoptosis induced by plitidepsin occurs via the mitochondrial (intrinsic) pathway. Sustained JNK activation occurs after Rac1 activation and downregulation of the phosphatase MKP-1, following depletion of glutathione, indicating a role for oxidative stress in plitidepsin-induced apoptosis [65]. Vascular endothelial growth factor (VEGF) secretion is inhibited in a human leukemia cell line, suggesting a possible effect of plitidepsin on angiogenesis [66]. Furthermore, the plitidepsin analogs PM01215 and PM02781 have been shown to inhibit angiogenesis in vivo as well as in vitro [67].

Tamandarins from a Brazilian ascidian have a very similar structure to didemnin B (Figure 2) and possess potent cytotoxic activity. Numerous studies on the chemical syntheses of these compounds and research into chemical modifications of tamandarins to find the molecular moieties important for biological activity have been reviewed [68]. Didemnin B has been found to be produced by the α-proteobacterium *Tistrella mobilis* obtained from marine sediment [69,70]. The biosynthetic gene cluster (*did*) encodes a 13-module hybrid nonribosomal peptide synthetase-polyketide synthase enzyme complex [70]. The discovery of this gene cluster may provide a solution to the supply problem and a route to the genetic engineering of new didemnin analogs.

### 4.2. Polyketides

**Figure 3:**
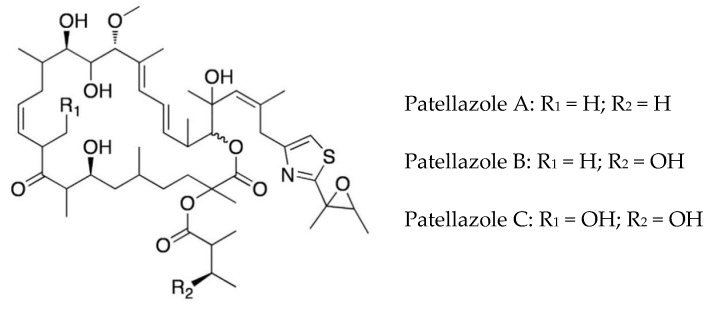
Structures of patellazoles A–C. Adapted from Kwan and Schmidt, [73]. (Creative Commons license: https://creativecommons.org/licenses/by/4.0/legalcode).

The highly cytotoxic patellazole A, which is thought to have a defensive role, is a polyketide-peptide hybrid made by α-proteobacterium Ca. *Endolissoclinum faulkneri* (Figure 3). This bacterium is only found in a subgroup of *Lissoclinum patella,* and its genome is extensively reduced, such that it could not live independently of the host. However, it maintains all the genes required for patellazole synthesis (*ptz* genes), providing evidence for an essential defensive role of these secondary metabolites in this symbiotic relationship [36,72,73]. The biosynthesis of patellazoles and other polyketides by the trans-AT polyketide synthases has been reviewed [74].

**Figure 4:**
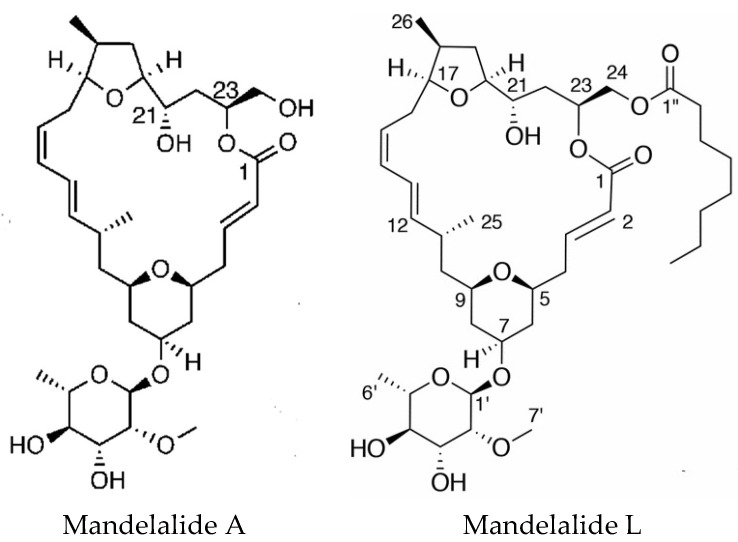
Structures of mandelalides A and L. Adapted with permission from (Nazari, M. et al. [78] and (Nazari, M. et al. [79]). Copyright 2018 American Chemical Society (Washington, DC, USA).

Mandelalides A–D are macrocyclic polyketides isolated from a new species *Lissoclinum mandelai* in South Africa [75]. Subsequently, the stereochemistry of mandelalide A was corrected [76] and the enantioselective total synthesis of mandelalide A and its ring-expanded macrolide isomer isomandelalide A was achieved [77]. Isomandelalide A exhibited unexpectedly high levels of activity being more potent than mandelalide B. The glycosylated mandelalides A and B are cytotoxic to neuroblastoma cells at low nanomolar concentrations [78]. New mandelalides, G–L, have been isolated allowing the study of structure–activity relationships, comparing the effects of monosaccharide and macrocyclic acylation on biological activity. The structures of mandelalides A and L are shown in Figure 4. The potent cytotoxicity of mandelalide A was found to be dependent on cell density with actively proliferating tumor cells at low density being relatively resistant to the compound. Mandelalides A and B inhibited mitochondrial function and induced caspase-dependent apoptotic cell death, due to inhibition of the mammalian ATP synthase complex V at concentrations of 30–100 nM, whereas the aglycosylated mandelamide C was much less potent. Cells with an oxidative phenotype were more likely to be inhibited. Cancer cells can shift their metabolism for ATP production from oxidative phosphorylation to aerobic glycolysis as nutrients become depleted, which could explain the effects of cell density [79].

Nine new natural products were isolated from *Didemnum molle* collected in Madagascar [80]. They are mollecarbamates A–D, which possess repeating o-carboxyphenethylamide units and a carbamate moiety; molleureas B–E, which contain tetra- and penta-repeating carboxyphenethylamide units and a urea bridge in different positions; and molledihydroisoquinolone, a cyclic form of o-carboxyphenethylamide. These metabolites were reported to be the only compounds known to contain ortho-carboxyphenethylamide derivatives in their skeleton. None of these compounds exhibited significant anti-viral or anti-bacterial activity.

The total synthesis of the cytotoxic polyketide Biselide E from an Okinawan Didemnid ascidian has recently been achieved [81].

## 5. Alkaloids

There are several structural families of alkaloids found in marine invertebrates, including indoles, pyrroles, pyrazines, quinolines, β-carbolines, and pyridoacridines. Marine alkaloids and synthetic analogs as important leads for anticancer drug development, have been reviewed [17], as have alkaloids derived specifically from ascidians [82]. An update on ascidian alkaloids and their modes of action, is provided in this section.

### 5.1. Quinoline Alkaloids

**Figure 5:**
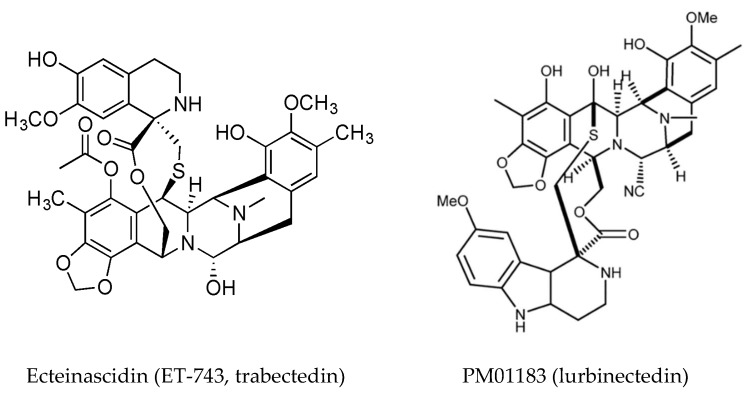
Structures of trabectedin (reproduced from [90]), and lurbinectedin, (reproduced from [17]).

Ecteinascidin (ET-743, trabectedin, Figure 5) is a tetrahydroisoquinoline alkaloid first isolated from the ascidian *Ecteinascidia turbinata* [83] and it has a known role in chemical defense [84]. It is approved as an anti-cancer drug in the US and Europe (commercialized by PharmMar) for soft tissue sarcomas and ovarian cancer under the trade name Yondelis^®^. The current status of trabectedin for the treatment of soft tissue sarcoma has been discussed [85]. Trabectedin binds DNA in the minor groove where it alkylates DNA residues, causing sequence specific alterations in DNA transcription and leading to DNA cleavage with subsequent apoptosis. A detailed description of its mechanism of action has been published [86]. Briefly, it forms an adduct with DNA leading to the formation of trabectedin/DNA/endonuclease ternary complexes, which on collision with replication forks, leading to double strand breaks which are repaired by homologous recombination. Binding of trabectedin to DNA interferes with transcription factors, other DNA binding proteins and repair pathways. Trabectedin modulates gene expression in a promoter- and gene-dependent manner, for example the expression of the Multidrug Resistance (*MDR-1*) gene that encodes P-glycoprotein is reduced and this could contribute to it anticancer activity [49,87]. In addition to its inhibition of trans-activated transcription factors, trabectedin affects the tumor microenvironment by induction of caspase-8 dependent apoptosis, specifically in monocytes and macrophages. The loss of these cells results in decreased expression of inflammatory cytokines [88,89]. Tumor associated macrophages have been implicated in progression and resistance of tumors to therapy and drive angiogenesis, thus targeting macrophages is an important aspect of the anti-tumor activity of trabectedin.

A detailed case study on the synthesis of trabectedin for pharmaceutical use is presented by Gomes and coworkers [90]. A synthetic analog of trabectedin, PM01183 (Lurbinectedin) [91] has a tetrahydro-β-carboline in the C subunit, as opposed to the tetrahydroisoquinoline present in trabectedin (Figure 5). They have a similar mode of action and comparable cytotoxicity. The tetrahydro β-carboline moiety protrudes from the minor groove of DNA and may interact directly with specific factors involved in DNA repair and transcription pathways [92]. Trabectedin- and lurbinectedin-adducts can interfere with the nucleotide excision repair (NER) machinery and cells deficient in NER are resistant to these compounds. NER is increased in cells which are resistant to cisplatin, providing a rationale for the combination of trabectedin or lurbinectedin with platinum drugs in clinical trials [93]. Lurbinectedin causes stalling of RNA polymerase II and inhibition of its phosphorylation, leading to its degradation by the proteasomal system, induction of DNA breaks and subsequently apoptosis [92]. Lurbinectedin, in combination with doxorubicin, showed remarkable activity in small-cell lung carcinoma and is currently in phase III trials for relapsed disease [94].

The DNA damage response initiated by trabectedin and lurbinectedin involves activation of two kinases, ataxia-telangiectasia mutated (ATM) and ataxia-telangiectasia related (ATR). Inhibition of both kinases simultaneously potentiates the cytotoxicity of these compounds providing a rationale for combining ATM and ATR inhibitors with the drugs to achieve maximal killing of tumor cells [95].

Trabectedin is made by microbial symbionts using the non-ribosomal peptide synthetase (NRPS) machinery. Using meta-genomic sequencing, the genes and 25 proteins in the biosynthetic pathway have been identified and characterized [96]. Sherman and colleagues have now taken out a patent on this pathway for the commercial production of NRPS-derived trabectedin [97]. This process will hopefully overcome the supply issues of currently used methods.

### 5.2. Pyridoacridine Alkaloids

The pyridoacridine alkaloids constitute the largest family of marine derived alkaloids, mainly from sponges and tunicates. The basic structural skeleton is 11*H*-pyrido[4,3,2-*mn*]acridine. The incredible chemical diversity and significant bioactivity of these compounds provide excellent targets for drug discovery for cytotoxic, anti-microbial, anti-parasitic, and antiviral agents [16,98]. These alkaloids have been classified according to the number of rings [99]. Pyridoacridines derived from ascidians are usually tetra- or penta-cyclic, possessing a functionalized alkylamine side chain but there are also some with 6, 7, or 8 rings [82]. This class of alkaloid are cytotoxic due to their core planar iminoquinone moiety that intercalates into DNA leading to breaks and they also inhibit topoisomerase II.

**Figure 6:**
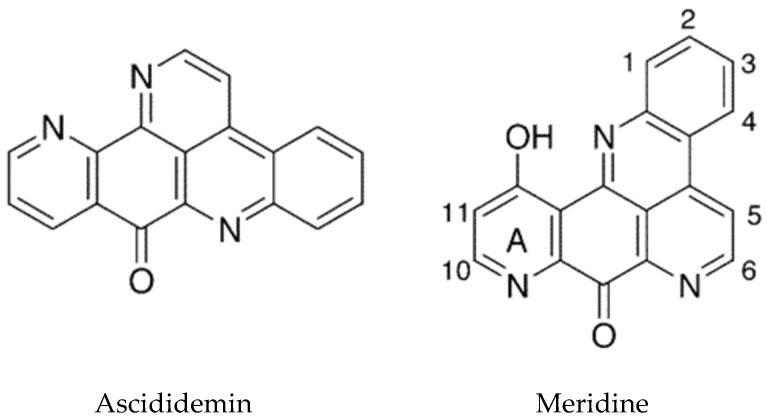
Pentacyclic pyridoacridine alkaloids. Adapted with permission from (Delfourne, E. et al. [104]). Copyright 2018 American Chemical Society (Washington, DC, USA).

The tetracyclic pyridoacridines are mostly derived from ascidians. Examples include cystodytins, styelsamines, diplamines, and varamines [16]. Cystodytin J and diplamine, which possess the iminoquinone portion, were found to be the best intercalators and inhibitors of topoisomerase II [99]. The pentacyclic structures include ascididemin (Figure 6), which intercalates into DNA preferentially at GC-rich sequences. Ascididemin has also been shown to induce DNA cleavage by a reactive oxygen species (ROS)-dependent mechanism, and to induce apoptosis in a mitochondrial dependent manner [99]. An additional effect of ascididemin is inhibition of telomerase activity. Elevated telomerase activity is one of the hallmarks of cancer [100]. Telomeres are protective repeat sequences at the end of chromosomes and shorten with each successive cell division in normal cells. In cancer cells, telomerase maintains the length of telomeric DNA promoting cell immortality, thus its inhibition is a useful approach for the development of anticancer drugs [101,102]. The G-rich strand of telomere DNA can fold into G-quadruplex structures which play an important role in telomere maintenance and cell cycle control via telomerase inhibition. Ascididemin as well as meridine (Figure 6) have been shown to stabilize G4 quadruplexes and thereby inhibit telomerase [103].

An in silico analysis of molecular docking of natural pyridoacridines with several anticancer targets was carried out [105]. The examination included the ascidian compounds meridine (*Amphicarpa meridiana*) and varamine A (*Lissoclinum vareau*). It was determined that the cyclin-dependent kinase, CDK6, was the most likely target for the pyridoacridines tested. Meridine (Figure 6) docking was predicted to be the most favorable, although these studies will need to be followed up experimentally. A recent review summarizes the progress that has been made in the synthetic chemistry of the pyridoacridine alkaloids [106].

### 5.3. Beta-Carboline Alkaloids

**Figure 7:**
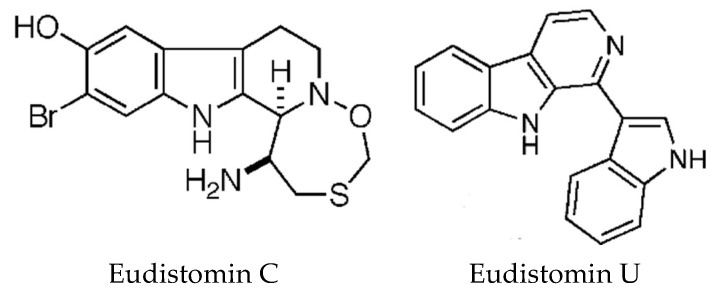
Beta-carboline alkaloids. Eudistomin C, reproduced from [111]. Eudistomin U, adapted with permission from (Panarese, J. and Waters S., [112]). Copyright 2018 American Chemical Society (Washington, DC, USA).

The carboline alkaloids are derived from tryptophan and found mostly in *Eudistoma* species [82]. All the β-carbolines are related biosynthetically in that tryptophan is coupled to a second amino acid, for example, eudistomin A is synthesised from tryptophan and glutamine. These compounds, originally isolated by Rinehart’s group [107], display a variety of biological activities with the oxathiazepino-eudistomins having strong antiviral properties [82]. DNA binding studies have been conducted with Eudistomin U (Figure 7) [108]. The structure of this alkaloid differs from other eudistomins, in that it contains an indole ring at the 1-position of the pyridine ring. Using several spectrophotometric techniques, it was shown that Eudistomin U binds DNA weakly with no sequence specificity and it was suggested that DNA binding may not be the mechanism of cytotoxicity. Eudistomin C (Figure 7), which has strong cytotoxic and antiviral properties, was recently reported to target the 40S ribosome and inhibit protein translation [109]. These authors identified yeast mutants resistant to EudiC and found mutations in the gene RPS14A which codes for the uS11 protein, a component of the 40S ribosome which interacts with eS1 and eS26 proteins that form the mRNA exit tunnel. Biotinylated EudiC pulled down us11 containing complexes from 40S ribosomes. Further investigations are required to completely understand its mechanism of action. The synthetic strategies and structure–activity relationships, of the β-carbolines have been reviewed by Kumar et al. [110].

### 5.4. Tyrosine and Phenylalanine Based Alkaloids

**Figure 8:**
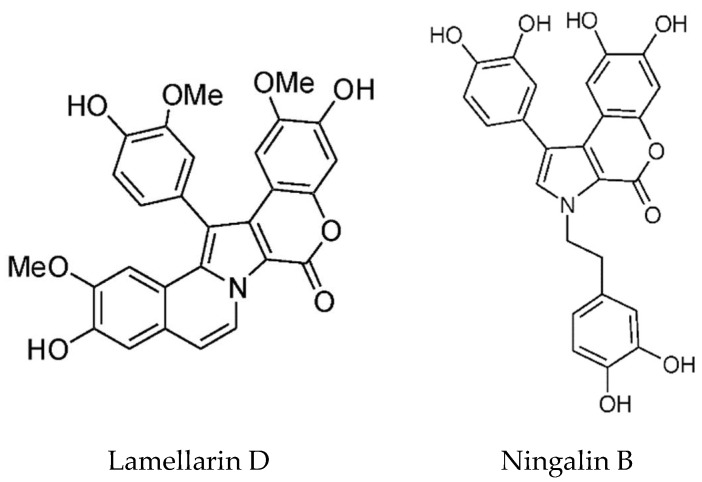
Structures of lamellarin D (reproduced from [117]), and ningalin B, (reproduced from [115]).

Lamellarins are DOPA and TOPA derived pyrrole alkaloids found in the prosobranch mollusk *Lamellaria* sp. as well as in the *Didemnid* ascidians and sponges on which the mollusks feed. There are two groups depending on whether the central pyrrole ring is fused or unfused [82]. Most of the lamellarins, and the related lukianols, polycitones, and ningalins possess a 3,4-diarylated pyrrole 2-carboxylic acid ester or amide moiety as the common structural subunit [113]. The majority of lamellarins are considerably cytotoxic with IC_50_ values in the nanomolar to micromolar range. The most cytotoxic is Lamellarin D (Figure 8) and structure–activity relationships have been determined [114]. Bailly has reviewed the anticancer properties of the lamellarins [115]. The main target of Lamellarin D is topoisomerase 1, with both the nuclear and mitochondrial forms being potently inhibited [115,116]. Topoisomerases cleave the DNA backbone to relax DNA supercoils and form transient enzyme-linked DNA breaks, which are referred to as cleavage complexes [116]. Lamellerin D, like camptothecin, traps the cleavage intermediates. It also directly acts on mitochondria, causing activation of Bax, release of apoptosis inducing factor (AIF) and caspase-3, and increase in ROS; however, mechanistic details are lacking. Lamellarin D, at sub-lethal doses, causes senescence which is dependent on topoisomerase 1 and ROS generation [117]. P388 cells were blocked in G2 phase, sometimes mutltinucleated with vacuolated cytoplasm, had elevated levels of p21 and stained positive for the senescence marker β-galactosidase. The source of ROS was determined not to be the mitochondria. The authors proposed a mechanism whereby topoisomerase inhibition led to limited DNA damage and in turn NOX-dependent ROS generation, p21 activation, cell cycle arrest, and then senescence.

The lamellarins have broad spectrum anticancer activity with multiple targets, including protein kinases and drug efflux pumps. ATP binding cassette (ABC) transporters such as P-glycoprotein (ABCB1), MDR-1 and ABCG2 (BCRP, breast cancer resistance protein) lead to drug efflux and multidrug resistance, which is a major problem in cancer treatment [115]. Natural products which inhibit these transporters to circumvent MDR have been reviewed [118]. Lamellarins I and K inhibit P-glycoprotein mediated drug efflux at nontoxic doses and are more potent than verapamil. Lamellarin D triacetate is the most potent P-gP inhibitor in the lamellarin class [119]. Structure–activity relationships with several lamellarins isolated from an Australian Didemnid ascidian and their synthetic derivatives have been investigated [120]. P-glycoprotein inhibitory activity was loosely correlated with higher levels of methylation on rings A and B. In view of their important biological activities, the lamellarins provide a platform for the synthesis of diverse analogs for drug discovery. The known synthetic routes to the lamellarin alkaloids published until 2014 have been reviewed [121]. Two papers describing the total synthesis of lamellarins D and H have recently been published [122,123].

Ningalin B (Figure 8) is an MDR reversal agent without cytotoxicity. Yang and coworkers synthesized 25 ningalin B derivatives, and evaluated their anti-P-glycoprotein activity [124]. Of these, compound 23, with dimethoxy groups at rings A and B and tri-substitution at ring C with ortho-bromo, meta-methoxy, and para-trimethoxybenzyloxy groups is the most potent inhibitor. It had an EC_50_ of 120–165 nM in reversing multidrug resistance and was shown to inhibit the transport activity of P-glycoprotein. Importantly for its potential clinical applications, it is not toxic. Based on their co-metabolite status and structural similarity, Plisson et al. [125] speculated that the ningalins and lamellarins share a common biosynthetic origin. They are assembled from a tyrosine and one to four substituted catechols, further modified by a limited number of cyclisations, dehydrations, oxidations and methylations. In addition, these compounds are inhibitors of several important kinases implicated in cancer and neurodegeneration—casein kinase 1d (CK1d), CDK5, and glycogen synthase kinase 3-β (GSK3-β). Ningalins C, D, and G were particularly noteworthy in this regard. The total synthesis of Ningalins D and G has been achieved [126].

Botryllamides are dehydrotyrosine derivatives isolated from styelid ascidians, *Botryllus* sp. Botryllamides A-J and a series of brominated tyrosine derivatives form a new class of selective inhibitors of ABCG2 (BCRP), in MDR cells with relatively low cytotoxicity [127]. Structure–activity relationships for ABCG2 binding have been determined [128]. Other ascidian metabolites which inhibit MDR are the patellamide cyanobactins [49].

### 5.5. Indole Based Alkaloids

**Figure 9:**
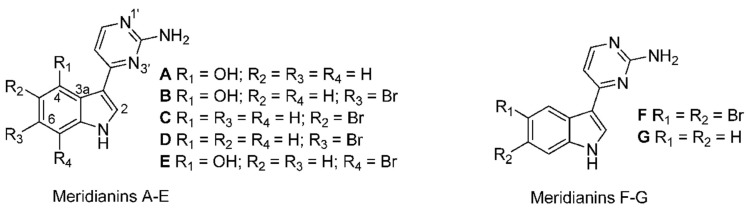
Structures of meridianins. Reproduced from [133].

Meridianins are indole alkaloids substituted at the C-3 position by 2-aminopyrimidine ring (Figure 9) and are potent protein kinase inhibitors, binding at the ATP binding pocket [129]. Meridianins, isolated from *Aplidium meridianum* in the East Weddell Sea in Antarctica, were shown to inhibit various protein kinases at low micromolar concentrations. These kinases include CDK, GSK-3β, CK1 and cyclic nucleotide-dependent kinases, which are important in cancer and neurodegenerative disease. Meridianin E was the most potent with selectivity for CDK1 and CDK5 [130]. A novel series of meridianin C derivatives substituted at the C-5 position have been prepared and structure–activity relationships (SAR) of the meridianin C core were determined [131]. One of the derivatives was shown to be a potent and selective inhibitor of the family of pim kinases—comprising pim-1, pim-2, and pim-3—with IC_50_ values in the nanomolar range. Pim kinases are often overexpressed in various cancers and play a role in cell cycle progression and signaling pathways initiated by cytokines and hormones. They are thus important targets for cancer therapy.

The same meridianin C derivatives, were tested for inhibition of lipid accumulation adipogenesis in differentiation of 3T3L1 preadipocytes into adipocytes [132]. It was determined that derivative 7b was the most potent. Inhibition occurred by downregulation of the expressions of CCAAT/enhancer-binding protein-α, (C/EBP-α), peroxisome proliferator-activated receptor-γ (PPAR-γ), fatty acid synthase (FAS), and the phosphorylation of STAT-3 (signal transducer and activator of transcription) and STAT-5 transcription factors. In addition, the expression of leptin mRNA was inhibited, suggesting a possible application for this compound in the treatment of obesity.

Mass spectrometric analysis of two antarctic ascidians led to the detection of 13 new meridianin analogs along with two dimers of meridianins B or E and A [133]. The search for new structures could provide more active forms for the development of potential drugs from this important family of kinase inhibiting alkaloids. Strategies taken to synthesize the meridianins and their derivatives have been reviewed [134].

A series of hybrid compounds of the CDK inhibitors, meridianin and variolin (from an Antarctic sponge) termed meriolins, (3-(pyrimidin-4-yl)-7-azaindoles), was synthesized to improve selectivity and efficiency, and several meriolins were tested for growth inhibitory and apoptotic activities in glioma cells [135]. The goal was to provide compounds with permeability to the blood brain barrier, less toxic effects on normal tissues and efficient combination with other chemotherapeutic agents. In vitro studies showed that meriolins 5 and 15 had potent antiproliferative activity on both astrocytes and glioblastoma multiforme (GBM) cells, and induced cell cycle arrest and promoted apoptosis. Meriolin 15 was also tested in vivo in a U87 glioblastoma xenograft model in nude mice. Administration of Meriolin 15 inhibited glioma cell proliferation, activated apoptosis and reduced the number of undifferentiated tumor stem cells. The authors proposed that meriolins inhibit multiple CDKs including CDK7/CDK9 thereby decreasing RNA polymerase II phosphorylation and leading to downregulation of the survival factor Mcl-1 and hence apoptosis. Further studies are in progress to find meriolin derivatives which are less toxic to normal cells.

Alkaloids of the staurosporine type have been frequently reported in *Eudistoma* species and 7-hydroxystaurosporine (UCN-01) has undergone clinical trials [18]. These will be discussed in more detail in Section 7 dealing with kinase inhibitors.

A bis-indole alkaloid eusynstelamide B from *Didemnum candidum* was investigated for its mechanism of action in prostate and breast cancer cell lines [136]. The compound causes G2 arrest and was identified as a novel non-intercalating topoisomerase II poison which activates DNA damage response pathways and induces double strand breaks. It shows comparable potency to the anti-cancer drug etoposide.

### 5.6. Other Alkaloids

**Figure 10:**
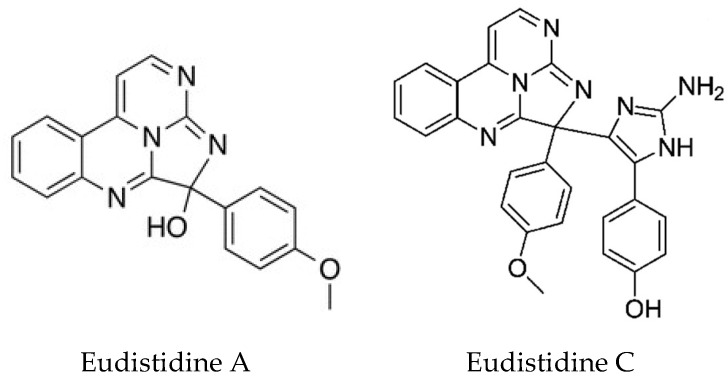
Structures of Eudistidines A and C. Eudistidine A; adapted with permission from (Chan, S. et al. [137]) Copyright 2018 American Chemical Society (Washington, DC, USA). Eudistidine C; adapted with permission from (Chan, S. et al. [138]) Copyright 2018 American Chemical Society (Washington, DC, USA).

Two novel metabolites Eudistidines A and B, unlike any other *Eudistoma* metabolites, have been isolated [137]. They represent a new structural class of alkaloids in which two pyrimidine rings and an imidazole ring are fused to generate a tetracyclic core. The biological activity of Eudistidine A is also very interesting, in that it can inhibit the interaction of the transcription factor HIF1 with the transcriptional co-activator protein p300. Tumors often grow under oxygen deprivation conditions. The HIF1-p300 complex is required for the transcription of hypoxia-responsive genes and represents an attractive therapeutic target for anticancer drugs. HIF1α is normally rapidly degraded. However, when oxygen tension is low, its levels accumulate in the nucleus where it binds to constitutively expressed HIF1β allowing the recruitment of p300 and expression of hypoxia responsive genes. Eudistidine A represents a new scaffold for the development of small molecule inhibitors of this interaction. A further paper by this group reported the synthesis of Eudistidine C which showed moderate inhibition of the HIF1/p300 interaction. Both Eudistidines A and C (Figure 10), also showed significant antimalarial activity against *Plasmodium falciparum,* including chloroquine resistant strains at low micromolar concentrations [138].

**Figure 11:**
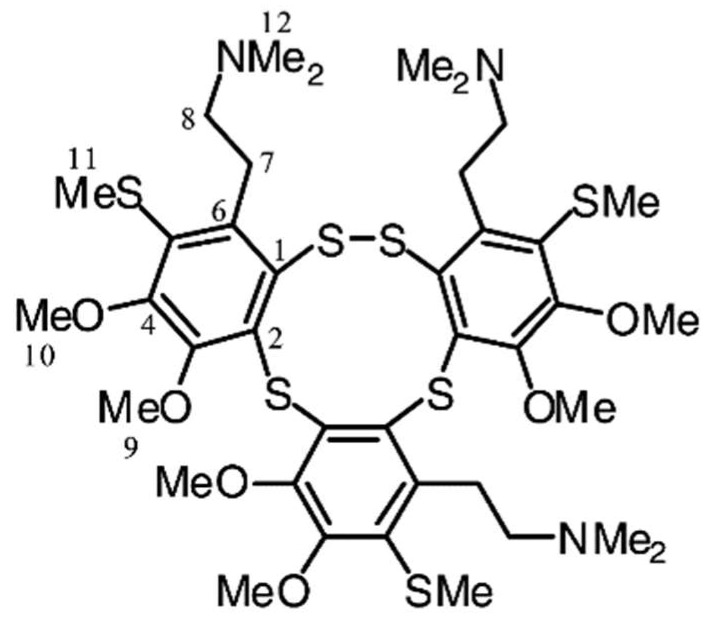
Lissoclibadin 1. Reproduced from [140].

Lissoclibadin 1 (Figure 11), a polysulfur aromatic alkaloid from *Lissoclinum* cf. *badium* with potent cytotoxic activity, induces caspase-dependent apoptosis in HCT-15 cells via the intrinsic pathway [139]. Importantly this study examined the in vivo anti-tumor efficacy in nude mice. It suppressed tumor growth without significant adverse effects, making it an ideal candidate for further investigation as an anticancer agent.

**Figure 12:**
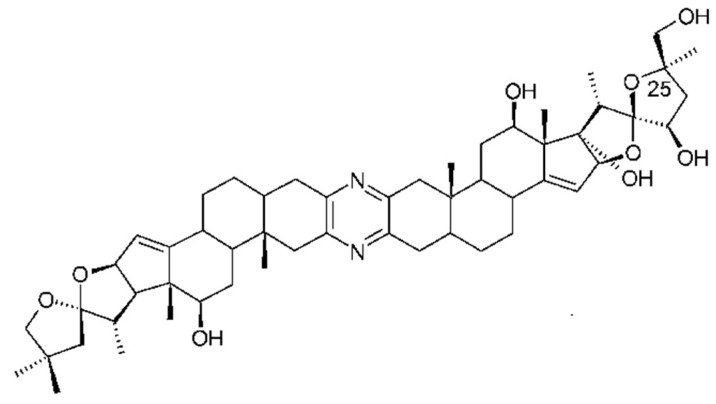
Structure of 25-epi-ritterostatin GN1N. Reproduced from [147].

Ritterazines originally isolated from *Riterella tokiada* (family *Polyclinidae*) [141] are dimeric steroidal pyrazine alkaloids closely related to cephalostatins from a marine worm (*Cephalodiscus gilchristi*), and microbial origin is suspected [142]. They have potent anti-tumor activity in the sub-nanomolar range. Ritterazine B is the most potent with an IC_50_ of 0.17nM. Structure–activity relationships of ritterazines and cephalostatins have been reviewed [17,82]. Ritterazines and cephalostatins share a common mode of action. The cellular target of the ritterostatin-cephalostatin hybrid, ritterostatin GN1N (Figure 12), was investigated using immunoaffinity fluorescent probes. It was shown to bind multiple heat shock proteins (Hsp70s) by immunoprecipitation. However, in the cell it is trafficked to the endoplasmic reticulum (ER) and binds predominantly to GRP78, activating the unfolded protein response and apoptosis [143]. Tahtamouni and coworkers [144] showed that two cephalostatin 1 analogs use the ER stress-induced apoptotic pathway. This involves caspase 4 activation, release of Smac/DIABLO, but not cytochrome C, from mitochondria and the phosphorylation of the eukaryotic initiation factor-2. The very limited natural sources of these potent compounds have necessitated chemical synthesis, a challenging task given their complex structures. A total synthesis of cephalostatin was achieved [145] and more recently a hybrid cephalostatin/ritterazine molecule [143] and a 12, 12′-azo-13, 13′-diepi-Ritterazine N analogue [146].

Three new alkaloids, stolonines A–C, were isolated from the ascidian *Cnemidocarpa stolonifera,* and chemically synthesized [148]. This is the first report of conjugates of taurine with 3-indoleglyoxylic acid, quinoline-2-carboxylic acid and β-carboline-3 carboxylic acid which are present in stolonines A–C respectively. Stolonines A and C were reported to induce apoptosis in PC3 cells. Four new iodobenzene containing dipeptides, a related bromotryptophan containing dipeptide, and an iodobenzene amine, have been isolated from the ascidian *Aplidium* sp. collected from Korean waters [149]. The compounds displayed moderate cytotoxicity and one, apliamide D, significantly inhibited the Na^+^/K^+^-ATPase.

## 6. Terpenoids and Quinones

Non alkaloid ascidian compounds of interest include the terpenoids and quinones which are discussed in this section.

### 6.1. Terpenoids

Terpenoids are derived from five-carbon isoprene units and classified according to the number of isoprene units. The lissoclimides, are a family of labdane diterpenoids bearing an unusual succinimide motif first isolated from *Lissoclinum voeltzkowi* Michaelsen [150]. Many of these compounds were reported to have potent cytotoxic activity against mammalian cancer cell lines. Könst and colleagues used short semi-synthesis and analogue-oriented synthetic approaches to produce a series of lissoclimide natural products and analogues for determination of structure–activity relationships (SAR) [151]. Toxicity was evaluated against the NCI’s 60 cancer cell line panel and was correlated to the protein synthesis inhibitory activity. Chlorolissoclimide was most potent with IC_50_ of 59nM, better than the marketed drug for chronic myelogenous leukemia (CML), Synribo^®^, the naturally occurring alkaloid homoharringtonine, which was first approved for treatment of CML by the FDA in 2012 [152]. The lissoclimides interfere with the elongation step of protein synthesis and prevent tRNA from exiting the ribosome, resulting in polysomal accumulation and eventual cell death. A crystallographic study of synthetic chlorolissoclimide bound to the eukaryotic 80S ribosome showed that it binds at the LSU ribosomal E-site in a manner similar to cycloheximide but with some novel interactions [151]. A high-yield short chemical synthesis of chlorolissoclimide using *N*-chloroamides to achieve site selective aliphatic C-H chlorination has been reported [153].

### 6.2. Quinones

**Figure 13:**
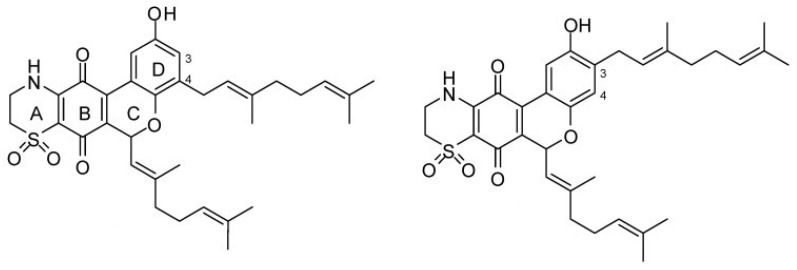
Thiaplidiaquinones A (**left**) and B (**right**). Reproduced from [156].

Quinones are derived from aromatic compounds and have a fully conjugated cyclic dione structure. The mechanism of action of four natural thiazinoquinones isolated from the ascidian *Aplidium conicum*—including Thiaplidiaquinone B, a prenylated benzoquinone—has been investigated [154]. This compound induces apoptosis in Jurkat cells by production of reactive oxygen species and depolarization of the mitochondrial membrane potential [155]. The quinones can intercalate between the base pairs of DNA and block DNA, RNA, and protein synthesis. The resulting stabilization of topoisomerase II binding leads to double strand breaks and the formation of ROS. The electrochemical response of the thiazinoquinones was measured in an aqueous environment and it was determined that on one-electron reduction, a semiquinone radical intermediate is formed [154]. This may be related to the cytotoxicity of these compounds, in that more redox reactions are initiated modifying DNA protein and lipids. The thiaplidiaquinones have also been studied in terms of their anti-malarial activity. They function by inhibiting the prenylating enzyme farnesyltransferase (FTase) in humans and parasites [156]. Extensive studies on the biological activity of the natural products thiaplidiaquinones A and B (Figure 13), as well as synthetic derivatives were performed [157]. The dioxothiazine regioisomers were found to be more potent having activities in the nanomolar range. SAR studies were carried out on synthesized prenyl and farnesyl analogs. The prenyl derivatives were the most potent in terms of inhibiting parasitic FTase but also the most cytotoxic, whereas the farnesyl series showed moderate activity with one analogue displaying minimal cytotoxicity. The geranyl series of compounds were the most potent at inhibiting FTase. However, none of the compounds exhibited good selectivity for parasitic vs. human FTase.

The isolation and structure elucidation of three isoquinoline quinones representing a novel type of tyrosine-based alkaloid from the ascidian *Ascidia virginea* Muller 1776 collected in Norway has been reported [158]. These compounds, named ascidines A-C, feature an intensely red chromophore and the authors speculated that they may be involved in defense since this particular ascidian is not damaged by feeders as opposed to other closely related ascidians in the same habitat. There is currently no information available on their cytotoxicity or mode of action.

## 7. Ascidian Compounds Affecting Signaling Pathways

Deregulation of signaling pathways is central to the development of cancer and neurodegenerative diseases. Several ascidian natural products affect these pathways, by inhibiting kinases and phosphatases and modulating neurotransmission.

### 7.1. Kinase Inhibitors

**Figure 14:**
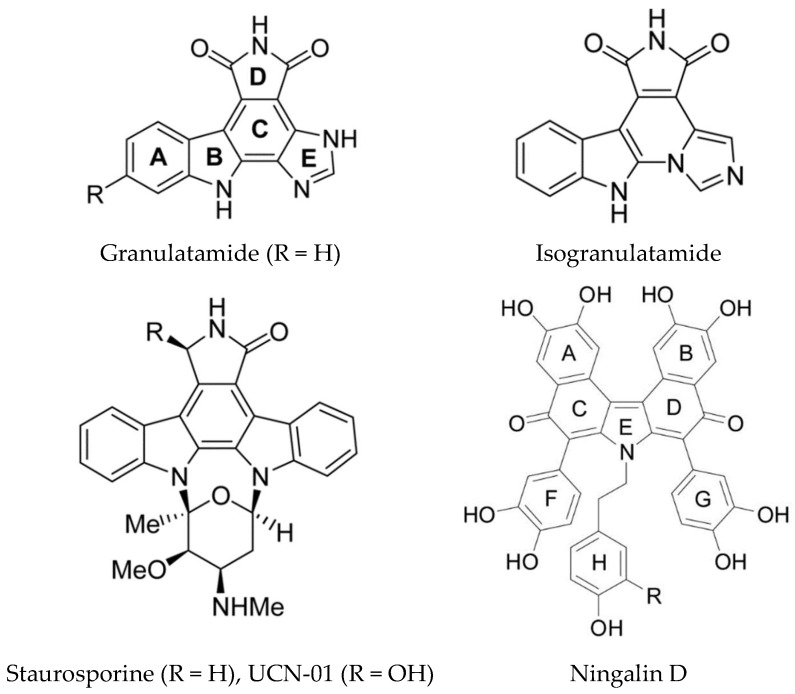
Selected kinase inhibitors from ascidians. Reproduced from [163], Ningalin from [115].

Staurosporine and related compounds inhibit several kinases including Akt (Protein Kinase B), Protein Kinase C (PKC), CDK, and Checkpoint Kinase 1 (Chk1). The clinical trials of 7-hydroxystaurosporine (UCN-01) revealed several toxicities due to the fact that several kinases are inhibited [18]. UCN-01 was shown to trigger the DNA damage response, cell cycle arrest and apoptosis in U2OS human osteosarcoma cells. Autophagy was also induced as a cell survival mechanism [159]. Two compounds isolated from the Brazilian ascidian *Didemnum granulatum* (granulatimide and isogranulatimide) (Figure 14) are inhibitors of Chk1 kinase, interacting with the ATP binding pocket [160]. Isogranulatimide has a unique indole/maleimide/ imidazole structure and the X-ray crystal structure of the Chk1-isogranulatimide complex has been determined. The aromatic pentacyclic planes of isogranulatimide resemble the aglycon part of the UCN-01 structure. A molecular docking based study was used to design potential new specific Chk1 inhibitors [161]. The same group also synthesized new amino or amido substituted analogs based on the granulatimide/isogranulatimide framework and examined their biological activity [162]. Two of the new compounds (where ring C is opened) were more potent than the parent compounds in inhibition of cell growth with IC_50_ values in the low micromolar range. However, this was shown not be due to Chk1 inhibition.

The polyandrocarpamines A and B isolated from the Fijian ascidian *Polyandrocarpa* sp. are 2-aminoimidazolone alkaloids. Several synthetic analogs of leucettamine B (a sponge natural product) with the 2-aminoimidazolone scaffold have been synthesized [164]. The researchers also tested a small library of sponge- and ascidian-derived 2-aminoimidazolone alkaloids for their kinase inhibitory activity against a panel of kinases (14 mammalian and 2 parasitic). The ascidian polyandrocarpamines A and B were shown to be potent inhibitors of cdc2-like kinases CLK1, CLK2, and dual-specificity tyrosine-regulated kinases (DYRK).

Dual-specificity tyrosine-regulated kinases, such as Dyrk1A, are over-expressed in several neurodegenerative diseases including Down syndrome and Alzheimer’s disease. A series of N-substituted meridianin derivatives were synthesized to further explore the SAR of meridianins for Dyrk1A inhibition and investigate their neuroprotective activity [129]. An N1-morpholinoyl substituted meridianin derivative, compound 26b, was identified as a promising inhibitor of Dyrk1A (IC_50_ 0.5 µM) with three- and four-fold selectivity for Dyrk1A with respect to Dyrk2 and Dyrk3. There was no cytotoxicity and it did not inhibit any of a panel of 15 other kinases tested. It also displayed promising neuroprotective activity in neuronal cells against glutamate induced neurotoxicity, indicating it is a useful lead for further development as an anti-Alzheimer’s disease (AD) agent. A key feature of the neurodegenerative pathology of this disease is accumulation of neurofibrillary tangles containing hyperphosphorylated tau protein [165]. Phosphorylation of tau regulates its binding to microtubules and the kinases involved in phosphorylating tau are GSK-3β, DYRK1A, CK1, and CLKs. Computer aided drug design has been used to computationally evaluate the inhibitory activity of meridianins A–G, against various protein kinases involved in AD [166]. Applying CADD to these kinases and meridianins A to G led to the identification of the important interactions with each complex, for example for GSK-3β, binding is thought to occur over the glycine rich loop. These studies could assist in the development of new analogs with improved inhibitory properties. Other ascidian alkaloids which inhibit GSK-3β, CK1, and CDK5 are ningalins C, D, and G (Figure 14) [167].

### 7.2. Acetylcholine Signaling Inhibitors

In addition to the kinase modulators discussed above, several other types of drug targets are being investigated in the search for treatments of AD [167]. Another feature of AD is reduction of nicotinic acetylcholine receptors in the cortex and hippocampus, with loss of cholinergic cells further contributing to cognitive decline [165]. Ascidian metabolites affecting the cholinergic neurotransmitter system include the acetylcholinesterase AChE inhibitors, Pulmonarins A and B, two dibrominated compounds from the ascidian *Synoicum pulmonaria* collected off the Norwegian coast [168]. Binding of Pulmonarin B is reversible and non-competitive with a K_i_ of 20 µM and there is no apparent cellular toxicity. It was suggested that binding occurs through electrostatic interactions at the peripheral anionic site (PAS) of the enzyme. This site is located on the surface of the protein at the entrance of the active site cleft [168].

The isolation and structural determination of two new β-carboline derivatives (irenecarbolines A and B) and a new purine derivative from a solitary ascidian *Cnemidocarpa irene* obtained from Japanese waters, has been reported. The β-carbolines are present in the blood of the ascidian and display significant anti AChE activity [169]. The function of these molecules in the blood is unknown. However, the authors noted that the cholinergic neuron of *Ciona intestinalis* larvae governs motor behavior and that the settlement of metamorphosing larvae was stimulated by acetylcholine.

The most abundant nicotinic acetylcholine receptors (nAChRs) in the central nervous system are 4βα2 heteromeric receptors and α7 homomeric receptors. Several natural products including the ascidian compounds, pibocin and varacin, were examined for binding to the nAChR, using computer modeling, binding studies and electrophysiological techniques [170]. Both compounds inhibited the binding of radiolabeled α-bungarotoxin and showed moderate activity towards mouse muscle and human α7 receptors. Pictamine from *Clavelina picta* is an antagonist at the nAChR, and Lepadin B is a potent blocker at two neuronal nicotinic acetylcholine receptors (α4β2 and α7) with IC_50_ values of 0.7–0.9 μM [171]. Since these compounds are antagonists rather than agonists, they would not be useful for treating AD. However, they may provide a useful tool for studying acetylcholine receptors. A new synthetic approach to (−)-lepadins A–C has been developed based on a stereocontrolled Diels–Alder reaction employing a chiral dienophile [172].

### 7.3. Phosphatase Inhibitors

Protein tyrosine phosphatases function to control cell signaling initiated at receptor tyrosine kinases. As such, they are an important molecular target to treat diseases such as cancer and diabetes. Recently, small molecule inhibitors for these enzymes are the focus of drug discovery endeavors [173]. Protein tyrosine phosphatase 1B (PTP1B) inhibitors from marine sources have been the subject of a recent review [174].

Two new merosesquiterpenes, Verruculides A and B, have been identified from the culture broth of the marine fungus *Penicillium verruculosum* TPU1311 originating from an ascidian [175]. These compounds were shown to inhibit protein tyrosine phosphatase 1B, an enzyme important in the negative regulation of insulin receptor signaling. PTP1B is a therapeutic target for obesity and type 2 diabetes. A new biphenyl ether derivative, along with the known benzophenone derivative, monodictyphenone, were isolated from an Indonesian ascidian-derived bacterium, *Penicillium albobiverticillium* TPU 1432 [176]. Both compounds were tested for inhibitory activity against three phosphatases, showing moderate activity with IC_50_ values of 20–43 µM.

**Figure 15:**
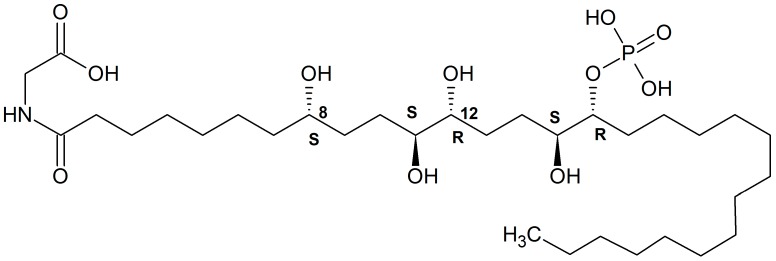
Phosphoeleganin structure. Redrawn from [178].

A new phosphorylated polyketide, phosphoeleganin (Figure 15), was isolated from a Mediterranean ascidian *Sidnyum elegans* [177]. It was also shown to inhibit protein tyrosine phosphatase 1B. The stereochemistry of this compound has recently been determined as 8S, 11S, 12 R, 15S, 16S [178].

## 8. Toxins Affecting the Cytoskeleton

The cytoskeleton controls cell attachment and movement, and performs vital functions in cell division. As such, it is an important target for anti-cancer drugs. A few ascidian toxins affect microtubules and actin filaments.

### 8.1. Tubulin

Agents which target microtubules, thereby inhibiting cell division, are widely used in cancer treatment, however their toxicity and neuropathy are often limiting. Marine natural products targeting microtubules are the subject of a recent review by Miller [179]. Ascidians produce three compounds which bind to tubulin, rigidin from *Eudistoma rigida*, vitilevuamide from *Didemnum cuculiferum* and diazonamide from *Diazona angulata.* These compounds destabilize microtubules. Diazonamide A is a complex cyclic peptide made by the non-ribosomal peptide synthetic pathway. It is a potent inhibitor of microtubule assembly and tubulin-dependent GTP hydrolysis [180]. It fails to inhibit the binding of vinblastine and colchicine to tubulin, indicating a unique binding site. A synthetic derivative of diazonamide, DZ-2384, exhibits potent anti-tumor activity towards multiple cancer types, while lacking neurotoxicity in rats at effective doses [181]. Using X-ray crystallographic and electron microscopy studies, it was shown to bind to the Vinca domain of tubulin in a distinct way. DZ-2384 differs from vinblastine by changing the pitch and curvature of tubulin protofilaments. Microtubule growth rate is slowed, however DZ-2384 increases microtubule rescue frequency compared with other Vinca alkaloids and this may explain its lesser neurotoxic effects. Several synthetic routes to Diazonamide A have previously been published. However, a formal total synthesis A by indole oxidative rearrangement has now been achieved [182].

### 8.2. Actin

Bistramide A (bisA, also known as bistratene A) is a spiroketal, first isolated from *Lissoclinum bistratum*. Spiroketals contains at least two oxacyclic rings, in which the oxygen atoms belonging to different rings share a common spiro-carbon atom. Bis A is a potent cytotoxin with IC_50_ in the nanomolar range. It has been shown to activate PKC-δ in HL-60 cells [183], however the primary mode of action appears to be binding to actin [184]. It sequesters G actin, inducing actin filament disassembly and inhibiting actin filament formation. Binding occurs at the barbed end and the ATP-binding domain. X-ray crystallographic studies of the bisA-actin complex showed that BisA has a unique binding site overlapping only slightly with that of other G-actin inhibitors [185]. Total internal reflection fluorescence TIRF microscopy was used to investigate actin filament dynamics in the presence of bisA. It has a unique mechanism of action in that it induces severing of actin filaments and covalent sequestration of monomeric actin via the enone portion of bisA. Importantly, the actin binding activity was not dependent on covalent modification [186]. This led to the rational design of compounds which target actin but do not react covalently. A simplified analog lacking the enone subunit reversibly bound actin and inhibited A549 non-small cell lung tumor cell growth in vitro and in vivo. There was no toxicity up to 50 mg/kg in a single intraperitoneal dose [187]. This contrasts with the toxicity of bisA itself, which has multiple effects on the central nervous system leading to paresthesia and loss of muscle tone [188]. The synthesis and preliminary biological evaluation of 35 stereoisomers of bisA has been reported [189]. One isomer displayed enhanced potency compared to the natural product in terms of actin binding and cellular cytotoxicity. Simplified analogs based on a hybrid bistramide-rhizopodin structure have been designed and synthesized and evaluated for their biological activity [190]. Most analogs displayed only moderate or no antiproliferative or actin binding activity. However, this work provides a basis for future development of more effective molecules. Early work on bisA showed that it induces cell cycle arrest, differentiation, inhibition of cytokinesis, and polyploidy in NSCLC-N6, HL-60, and melanoma cell lines [summarized in 183]. In HL-60 cells treated with bisA, the normally floating cells attach to the substratum and put out processes. In contrast, fibroblasts, which are normally attached, lift off as their actin filaments dissociate. Several proteins are phosphorylated in the presence of bisA in HL-60 and melanoma cells, however the signaling pathway induced by bisA is not known. The literature from 2011 to July 2017 on the bioactivity, biosynthesis, and chemical synthesis of structurally diverse spiroketals—including a few ascidian compounds—has been reviewed [191].

## 9. Conclusions and Future Perspectives

Ascidians have provided a treasure trove of interesting biologically active compounds and much progress has been made in recent years. Table 1 lists the major classes of compounds discussed in this review with their biological activity and molecular targets. There are several major challenges in the pipeline from natural product discovery to therapeutic drugs, including the need for sufficient supply of compounds and a lack of knowledge of the biosynthetic pathways or even the responsible organism (symbiotic bacterium or host ascidian) and mechanism of action. Taxonomic identification of ascidians by morphology is also a problem, due to the shortage of expert taxonomists. This can be overcome with DNA barcoding, recently applied to *Lissoclinum fragile* and four Indian ascidians by sequencing a short segment of mitochondrial DNA coding for subunit 1 of cytochrome C oxidase [192,193].

Many potential drugs fail in clinical trials. Eastman argues that this high failure rate could be much reduced with better preclinical testing [194]. For example, many of the cell viability assays used do not actually measure viability i.e. live vs. dead cells. Instead many assays use growth inhibition as an endpoint, and fewer cells does not actually mean that they have lost viability [194]. Due to lack of biological material, the biological activity and mechanism of action of many compounds remains to be thoroughly investigated. Using ET-743 (trabectedin) as an example, Gomes and coworkers have reviewed the strategies being used to tackle this supply problem, including marine invertebrate aquaculture, invertebrate, and symbiont cell culture, culture-independent strategies, total chemical synthesis, and semi-synthesis, and several hybrid strategies [90]. Similarly, Newman has reviewed four natural product-derived drugs, including ET-743, from the point of view of how supply problems have been overcome [195]. Initial studies by PharmaMar were done with ET-743 isolated from massive collections in the Caribbean, then with a semi-synthetic method starting with the bacterial product safracin B. Identification of the genomic biosynthetic pathway and the probable producing organism, γ-proteobacterium *Candidatus Endoecteinascidia frumentensis* (AY054370), opens the way for a biotechnological approach [96,97,196,197]. The microorganism has not been cultured to date, however the genes encoding the biosynthetic pathway could potentially be expressed in a heterologous host. Zhang and colleagues have reviewed the advanced tools that are currently available to maximize drug discovery, from chemical analysis, metabolomics, high throughput screening, to metagenomics, genome mining, and biotechnological synthesis of natural products [198]. Although challenging, chemical synthesis of these often very complex molecules also provides a means of producing sufficient compound, as well as access to potentially active derivatives not found in nature. Some of these have also been highlighted in this review.

## Figures and Tables

**Table 1:** Ascidian toxins and their mode of action.

Compound	Ascidian Source	Compound Class	Biological Activity	Molecular Target(s)	References
Ascididemin	*Didemnum* sp.	Pyridoacridine alkaloid	Cytotoxic	DNA intercalation, stabilizes G4 quadriplexes and inhibits telomerase	[99,103]
Bistramides	*Lissoclinum bistratum*	Spiroketal	Cytotoxic, induces protein phosphorylation	Actin filaments	[183,184,185,186,187,188]
Bistratamides	*Lissoclinum bistratum*	Cyanobactins	Cytotoxic, Metal binding	unknown	[45]
Botryllamides	*Botryllus* sp.	Brominated tyrosine derivatives	MDR reversal	ABCG2	[127,128]
Diazonamide A	*Diazona angulata*	Cyclic peptide	Cytotoxic	Microtubules	[179,180,181]
Didemnin B	*Tridemnum solidum*	Cyclic depsipeptide	Cytotoxic, Inhibition of protein translation, immunosuppressive, antiviral	eEF1A1PPT1	[57,58,59]
Eudistidines	*Eudistoma* sp.	Novel alkaloids	Inhibition of protein-protein interaction, anti-malarial	HIF1-p300	[137,138]
Eudistomin C	*Eudistoma* sp.	β-Carboline alkaloid	Cytotoxic, anti-viral, Inhibition of protein translation	us11 protein on 40S ribosome	[109]
Euseynstelamide B	*Didemnum candidum*	Bis-indole alkaloid	Cytotoxic, causing G2 arrest	Topoisomerase II	[136]
Granulatimides	*Didemnum granulatum*	Alkaloids	Kinase Inhibition	Chk1 (kinase)	[160,161,162]
Irenecarbolines	*Cnemidocarpa irene*	β-carbolines	Enhancement of cholinergic neurotransmission	AChE inhibitors	[169]
Lamellarins	*Didemnum* sp.	DOPA/TOPA derived pyrrole alkaloids	Cytotoxic	Multiple targets – Topoisomerase 1, Kinases, Drug efflux pumps e.g. MDR-1, P-glycoprotein	[114,115,116,117,118,119]
Lissoclibadins	*Lissoclinum* cf *badium*	Polysulfur aromatic alkaloids	Cytotoxic, anti-tumor in mice	unknown	[139]
Lissoclimides	*Lissoclinum voeltzkowi Michaelsen*	Labdane diterpenoids	Cytotoxic, inhibition of elongation step of protein synthesis	LSU Ribosomal E-site	[151]
Lissoclinamides	*Lissoclinum patella*	Cyanobactins	Cytotoxic, Metal Binding	unknown	[51]
Mandelalides A & B	*Lissoclinum mandelai*	Polyketides	Cytotoxic	ATP synthase complex V	[78,79]
Meridianins	*Aplidium meridianum*	Indole alkaloids	Kinase inhibition	GSK-3β, CK1, CDKs	[129,130,131]
Meridine	*Amphicarpa meridiana*	Pyridoacridine alkaloid	Cytotoxic	DNA, stabilizes G4 quadriplexes and inhibits telomerase	[104]
Ningalins	*Didemnum* sp.	DOPA/TOPA derived pyrrole alkaloids	MDR reversal, kinase inhibition	MDR-1, P-glycoprotein	[124,125,167]
Patellamides	*Lissoclinum patella*	Cyanobactins	Cytotoxic, metal binding	MDR-1, others unknown	[46,47,49]
Patellazoles A–C	*Lissoclinum patella*	Polyketides	Cytotoxic, chemical defense	unknown	[36,72,73]
Phosphoeleganin	*Sidnyum elegans*	Polyketide	Phosphatase inhibition	PTP1B	[177,178]
Pibocin, Varacin, Pictamine, Lepadin	*Eudistoma* sp. *Lissoclinum* sp. *Clavelina picta* *Clavelina lepadiformis*	Ergoline alkaloid Benzopentathiepin Quinolizidine alkaloid Decahydroquinoline alkaloid	Inhibition of cholinergic neurotransmission Inhibition of cholinergic neurotransmission	nAChR antagonistsn AChR antagonists	[171]
Plitidepsin (dehydrodidemnin B) Aplidin^®^	*Aplidia albicans*	Cyclic depsipeptide	Anticancer drug	eEF1A2	[63]
Polyandrocarpamines A & B	*Polyandrocarpa* sp.	2-aminoimidazolone alkaloid	Kinase inhibition	CLK1, CLK2, DYRK	[164]
Pulmonarins A & B	*Synoicum pulmonaria*	Dibrominated tyrosine derivatives	Enhancement of cholinergic neurotransmission	AChE inhibitors	[168]
Ritterazines	*Riterella tokiada*	Dimeric steroidal pyrazine alkaloids	Cytotoxic	Hsp70s, GRP78	[17,82,143]
Tamandarins	*Unidentified Brazilian species*	Cyclic depsipeptides, closely related to didemnin B	Cytotoxic	Unknown but may be similar to didemnin B	[68]
Thiaplidiaquinones	*Aplidium conicum*	Thiazinoquinones	Cytotoxic, anti-malarial	DNA, stabilizes topoisomerase II, ROS generation. FTase	[154,155,156,157]
Trabectidin (ET-473) Yondelis^®^	*Ecteinascidia turbinata*	Tetrahydroisoquinoline alkaloid	Anticancer drug, Induces apoptosis in tumor associated macrophages	DNA, minor groove, interference with transcription factors and DNA repair proteins	[49,86,87,88,89]
UCN-01 (7-hydroxystaurosporine)	*Eudistoma* sp.	Alkaloid	Kinase inhibition	Multiple kinases	[159]

## References

[1] Lambert C.C. (2005). Historical introduction, overview, and reproductive biology of the protochordates. Can. J. Zool..

[2] Lambert G. (2005). Ecology and natural history of the protochordates. Can. J. Zool..

[3] Shenkar N., Swalla B.J. (2011). Global diversity of Ascidiacea. PLoS ONE.

[4] Shenkar N., Gittenberger A., Lambert G., Rius M., Moreira Da Rocha R., Swalla B.J., Turon X. (2018). Ascidiacea World Database. http://www.marinespecies.org/ascidiacea.

[5] Bellante A., Piazzese D., Cataldo S., Parisi M.G., Cammarata M. (2016). Evaluation and comparison of trace metal accumulation in different tissues of potential bioindicator organisms: Macrobenthic filter feeders *Styela plicata*, *Sabella spallanzanii*, and *Mytilus galloprovincialis*. Environ. Toxicol. Chem..

[6] Dumollard R., Gazo I.D.L., Gomes I., Besnardeau L., McDougall A. (2017). Ascidians: An Emerging Marine Model for Drug Discovery and Screening. Curr. Top. Med. Chem..

[7] Ueki T., Yamaguchi N., Isago Y., Tanahashi H. (2015). Vanadium accumulation in ascidians: A system overview. Coord. Chem. Rev..

[8] Palanisamy S.K., Thomas O.P., McCormack G.P. (2018). Bio-invasive ascidians in Ireland: A threat for the shellfish industry but also a source of high added value products. Bioengineered.

[9] Palanisamy S.K., Trisciuoglio D., Zwergel C., Del Bufalo D., Mai A. (2017). Metabolite profiling of ascidian *Styela plicata* using LC–MS with multivariate statistical analysis and their antitumor activity. J. Enzyme Inhib. Med. Chem..

[10] Lee Y., Phat C., Hong S.C. (2017). Structural diversity of marine cyclic peptides and their molecular mechanisms for anticancer, antibacterial, antifungal, and other clinical applications. Peptides.

[11] Negi B., Kumar D.S., Rawat D. (2017). Marine peptides as anticancer agents: A remedy to mankind by nature. Curr. Protein Pept. Sci..

[12] Kang H.K., Choi M.C., Seo C.H., Park Y. (2018). Therapeutic Properties and Biological Benefits of Marine-Derived Anticancer Peptides. Int. J. Mol. Sci..

[13] Fang W.Y., Dahiya R., Qin H.L., Mourya R., Maharaj S. (2016). Natural proline-rich cyclopolypeptides from marine organisms: Chemistry, synthetic methodologies and biological status. Mar. Drugs.

[14] Gogineni V., Hamann M.T. (2018). Marine natural product peptides with therapeutic potential: Chemistry, biosynthesis, and pharmacology. Biochim. Biophys. Acta (BBA)-Gen. Subj..

[15] Ruiz-Torres V., Encinar J.A., Herranz-López M., Pérez-Sánchez A., Galiano V., Barrajón-Catalán E., Mico V. (2017). An updated review on marine anticancer compounds: The use of virtual screening for the discovery of small-molecule cancer drugs. Molecules.

[16] Ibrahim S.R., Mohamed G.A. (2016). Marine pyridoacridine alkaloids: Biosynthesis and biological activities. Chem. Biodivers..

[17] Imperatore C., Aiello A., D’Aniello F., Senese M., Menna M. (2014). Alkaloids from marine invertebrates as important leads for anticancer drugs discovery and development. Molecules.

[18] Palanisamy S.K., Rajendran N.M., Marino A. (2017). Natural Products Diversity of Marine Ascidians (Tunicates; Ascidiacea) and Successful Drugs in Clinical Development. Nat. Prod. Bioprospect..

[19] Arumugam V., Venkatesan M., Ramachandran S., Sundaresan U. (2018). Bioactive Peptides from Marine Ascidians and Future Drug Development—A Review. Int. J. Pept. Res. Ther..

[20] Cooper E.L., Albert R. (2015). Tunicates: A vertebrate ancestral source of antitumor compounds. Handbook of Anticancer Drugs from Marine Origin.

[21] Agrawal S., Adholeya A., Deshmukh S.K. (2016). The Pharmacological Potential of Non-ribosomal Peptides from Marine Sponge and Tunicates. Front. Pharmacol..

[22] Mayer C.A. The Global Marine Pharmaceuticals Pipeline. http://marinepharmacology.midwestern.edu/clinPipeline.htm.

[23] Donia M.S., Fricke W.F., Partensky F., Cox J., Elshahawi S.I., White J.R., Phillippy A.M., Schatz M.C., Piel J., Haygood M.G. (2011). Complex microbiome underlying secondary and primary metabolism in the tunicate-*Prochloron* symbiosis. Proc. Natl. Acad. of Sci. USA.

[24] Schmidt E.W., Donia M.S., McIntosh J.A., Fricke W.F., Ravel J. (2012). Origin and variation of tunicate secondary metabolites. J. Nat. Prod..

[25] Chen L., Fu C., Wang G. (2017). Microbial diversity associated with ascidians: A review of research methods and application. Symbiosis.

[26] Schmidt E.W. (2015). The secret to a successful relationship: Lasting chemistry between ascidians and their symbiotic bacteria. Invertebr. Biol..

[27] Tianero M.D., Kwan J.C., Wyche T.P., Presson A.P., Koch M., Barrows L.R., Bugni T.S., Schmidt E.W. (2015). Species specificity of symbiosis and secondary metabolism in ascidians. ISME J..

[28] Evans J.S., Erwin P.M., Shenkar N., López-Legentil S. (2017). Introduced ascidians harbor highly diverse and host-specific symbiotic microbial assemblages. Sci. Rep..

[29] Buedenbender L., Carroll A.R., Ekins M., Kurtböke D.İ. (2017). Taxonomic and Metabolite Diversity of *Actinomycetes* Associated with Three Australian Ascidians. Diversity.

[30] Donia M.S., Ravel J., Schmidt E.W. (2008). A global assembly line for cyanobactins. Nat. Chem. Biol..

[31] Sivonen K., Leikoski N., Fewer D.P., Jokela J. (2010). Cyanobactins—Ribosomal cyclic peptides produced by cyanobacteria. Appl. Microbiol. Biotechnol..

[32] Behrendt L., Raina J.B., Lutz A., Kot W., Albertsen M., Halkjær-Nielsen P., Sørensen S.J., Larkum A.W., Kühl M. (2018). In situ metabolomic-and transcriptomic-profiling of the host-associated cyanobacteria *Prochloron* and *Acaryochloris marina*. ISME J..

[33] Hirose E., Neilan B.A., Schmidt E.W., Murakami A., Gault P.M., Gault P.M., Marler H.J. (2007). Enigmatic life and evolution of *Prochloron* and related cyanobacteria inhabiting colonial ascidians. Handbook on Cyanobacteria: Biochemistry, Biotechnology and Applications.

[34] Hirose E. (2015). Ascidian photosymbiosis: Diversity of cyanobacterial transmission during embryogenesis. Genesis.

[35] Martins J., Vasconcelos V. (2015). Cyanobactins from cyanobacteria: Current genetic and chemical state of knowledge. Mar. Drugs.

[36] Morita M., Schmidt E.W. (2018). Parallel lives of symbionts and hosts: Chemical mutualism in marine animals. Nat. Prod. Rep..

[37] Schmidt E.W., Nelson J.T., Rasko D.A., Sudek S., Eisen J.A., Haygood M.G., Ravel J. (2005). Patellamide A and C biosynthesis by a microcin-like pathway in *Prochloron didemni*, the cyanobacterial symbiont of *Lissoclinum patella*. Proc. Natl. Acad. Sci. USA.

[38] Sardar D., Schmidt E.W. (2016). Combinatorial biosynthesis of RiPPs: Docking with marine life. Curr. Opin. Chem. Biol..

[39] Czekster C.M., Ge Y., Naismith J.H. (2016). Mechanisms of cyanobactin biosynthesis. Curr. Opin. Chem. Biol..

[40] Koehnke J., Bent A.F., Houssen W.E., Mann G., Jaspars M., Naismith J.H. (2014). The structural biology of patellamide biosynthesis. Curr. Opin. Struct. Biol..

[41] Lin Z., Torres J.P., Tianero M.D., Kwan J.C., Schmidt E.W. (2016). Origin of chemical diversity in *Prochloron*-tunicate symbiosis. Appl. Environ. Microbiol..

[42] Tianero M.D., Pierce E., Raghuraman S., Sardar D., McIntosh J.A., Heemstra J.R., Schonrock Z., Covington B.C., Maschek J.A., Cox J.E. (2016). Metabolic model for diversity-generating biosynthesis. Proc. Natl. Acad. Sci. USA.

[43] Adaba R.I., Mann G., Raab A., Houssen W.E., McEwan A.R., Thomas L., Tabudravu J., Naismith J.H., Jaspars M. (2016). Accurate quantification of modified cyclic peptides without the need for authentic standards. Tetrahedron.

[44] Bertram A., Pattenden G. (2007). Marine metabolites: Metal binding and metal complexes of azole-based cyclic peptides of marine origin. Nat. Prod. Rep..

[45] Urda C., Fernández R., Rodríguez J., Pérez M., Jiménez C., Cuevas C. (2017). Bistratamides M and N, oxazole-thiazole containing cyclic hexapeptides isolated from *Lissoclinum bistratum* interaction of zinc (II) with bistratamide K. Mar. Drugs.

[46] Comba P., Dovalil N., Gahan L.R., Hanson G.R., Westphal M. (2014). Cyclic peptide marine metabolites and Cu II. Dalton Trans..

[47] Comba P., Gahan L.R., Hanson G.R., Maeder M., Westphal M. (2014). Carbonic anhydrase activity of dinuclear CuII complexes with patellamide model ligands. Dalton Trans..

[48] Comba P., Eisenschmidt A., Gahan L.R., Herten D.P., Nette G., Schenk G., Seefeld M. (2017). Is CuII Coordinated to Patellamides inside Prochloron Cells?. Chem. Eur. J..

[49] Lopez D., Martinez-Luis S. (2014). Marine natural products with P-glycoprotein inhibitor properties. Mar. Drugs.

[50] Wipf P., Uto Y. (2000). Total synthesis and revision of stereochemistry of the marine metabolite trunkamide A. J. Org. Chem..

[51] Xie S., Savchenko A.I., Kerscher M., Grange R.L., Krenske E.H., Harmer J.R., Bauer M.J., Broit N., Watters D.J., Boyle B.M. (2018). Heteratom-Interchanged isomers of Lissoclinamide 5: Copper(II) complexation, halide binding and biological activity. Eur. J. Org. Chem..

[52] Salib M.N., Molinski T.F. (2017). Cyclic Hexapeptide Dimers, Antatollamides A and B, from the Ascidian *Didemnum molle*. A Tryptophan-Derived Auxiliary for l-and d-Amino Acid Assignments. J. Org. Chem..

[53] Taevernier L., Wynendaele E., Gevaert B., De Spiegeleer B. (2017). Chemical classification of cyclic depsipeptides. Curr. Protein Peptide Sci..

[54] Süssmuth R.D., Mainz A. (2017). Nonribosomal Peptide Synthesis—Principles and Prospects. Angew. Chem. Int. Ed..

[55] Amoutzias G.D., Chaliotis A., Mossialos D. (2016). Discovery strategies of bioactive compounds synthesized by nonribosomal peptide synthetases and type-I polyketide synthases derived from marine microbiomes. Mar. Drugs.

[56] Rinehart K.L., Gloer J.B., Cook J.C., Mizsak S.A., Scahill T.A. (1981). Structures of the didemnins, antiviral and cytotoxic depsipeptides from a Caribbean tunicate. J. Am. Chem. Soc..

[57] Marco E., Martín-Santamaría S., Cuevas C., Gago F. (2004). Structural basis for the binding of didemnins to human elongation factor eEF1A and rationale for the potent antitumor activity of these marine natural products. J. Med. Chem..

[58] Potts M.B., McMillan E.A., Rosales T.I., Kim H.S., Ou Y.H., Toombs J.E., Brekken R.A., Minden M.D., MacMillan J.B., White M.A. (2015). Mode of action and pharmacogenomic biomarkers for exceptional responders to didemnin B. Nat. Chem. Biol..

[59] Thell K., Hellinger R., Schabbauer G., Gruber C.W. (2014). Immunosuppressive peptides and their therapeutic applications. Drug Discov. Today.

[60] Alonso-Álvarez S., Pardal E., Sánchez-Nieto D., Navarro M., Caballero M.D., Mateos M.V., Martín A. (2017). Plitidepsin: Design, development, and potential place in therapy. Drug Des. Dev. Ther..

[61] US National Library of Medicine. www.clinicaltrials.gov.

[62] PharmaMar PharmaMar Has Requested the Process of Re-Examination for Aplidin® from the EMA. https://www.pharmamar.com/wp-content/uploads/2018/01/PR_PharmaMar-has-requested-the-process-of-re-examination-for-Aplidin%C2%AE-from-the-EMA.pdf.

[63] Losada A., Muñoz-Alonso M.J., García C., Sánchez-Murcia P.A., Martínez-Leal J.F., Domínguez J.M., Lillo M.P., Gago F., Galmarini C.M. (2016). Translation elongation factor eEF1A2 is a novel anticancer target for the marine natural product plitidepsin. Sci. Rep..

[64] Muñoz-Alonso M.J., González-Santiago L., Zarich N., Martínez T., Alvarez E., Rojas J.M., Muñoz A. (2008). Plitidepsin has a dual effect inhibiting cell cycle and inducing apoptosis via Rac1/c-Jun NH2-terminal kinase activation in human melanoma cells. J. Pharmacol. Exp. Ther..

[65] González-Santiago L., Suárez Y., Zarich N., Muñoz-Alonso M.J., Cuadrado A., Martinez T., Goya L., Iradi A., Sáez-Tormo G., Maier J.V. (2006). Aplidin® induces JNK-dependent apoptosis in human breast cancer cells via alteration of glutathione homeostasis, Rac1 GTPase activation, and MKP-1 phosphatase downregulation. Cell Death Differ..

[66] Broggini M., Marchini S.V., Galliera E., Borsotti P., Taraboletti G., Erba E., Sironi M., Jimeno J., Faircloth G.T., Giavazzi R. (2003). Aplidine, a new anticancer agent of marine origin, inhibits vascular endothelial growth factor (VEGF) secretion and blocks VEGF-VEGFR-1 (flt-1) autocrine loop in human leukemia cells MOLT-4. Leukemia.

[67] Borjan B., Steiner N., Karbon S., Kern J., Francesch A., Hermann M., Willenbacher W., Gunsilius E., Untergasser G. (2015). The Aplidin analogs PM01215 and PM02781 inhibit angiogenesis in vitro and in vivo. BMC Cancer.

[68] Lee J., Currano J.N., Carroll P.J., Joullié M.M. (2012). Didemnins, tamandarins and related natural products. Nat. Prod. Rep..

[69] Tsukimoto M., Nagaoka M., Shishido Y., Fujimoto J., Nishisaka F., Matsumoto S., Harunari E., Imada C., Matsuzaki T. (2011). Bacterial production of the tunicate-derived antitumor cyclic depsipeptide didemnin B. J. Nat. Prod..

[70] Xu Y., Kersten R.D., Nam S.J., Lu L., Al-Suwailem A.M., Zheng H., Fenical W., Dorrestein P.C., Moore B.S., Qian P.Y. (2012). Bacterial biosynthesis and maturation of the didemnin anti-cancer agents. J. Am. Chem. Soc..

[71] Adrio J., Cuevas C., Manzanares I., Joullié M.M. (2007). Total synthesis and biological evaluation of tamandarin B analogues. J. Org. Chem..

[72] Kwan J.C., Donia M.S., Han A.W., Hirose E., Haygood M.G., Schmidt E.W. (2012). Genome streamlining and chemical defense in a coral reef symbiosis. Proc. Natl. Acad. Sci. USA.

[73] Kwan J.C., Schmidt E.W. (2013). Bacterial endosymbiosis in a chordate host: Long-term co-evolution and conservation of secondary metabolism. PLoS ONE.

[74] Helfrich E.J., Piel J. (2016). Biosynthesis of polyketides by trans-AT polyketide synthases. Nat. Prod. Rep..

[75] Sikorska J., Hau A.M., Anklin C., Parker-Nance S., Davies-Coleman M.T., Ishmael J.E., McPhail K.L. (2012). Mandelalides A–D, cytotoxic macrolides from a new *Lissoclinum* species of South African tunicate. J. Org. Chem..

[76] Lei H., Yan J., Yu J., Liu Y., Wang Z., Xu Z., Ye T. (2014). Total synthesis and stereochemical reassignment of mandelalide A. Angew. Chem. Int. Ed..

[77] Veerasamy N., Ghosh A., Li J., Watanabe K., Serrill J.D., Ishmael J.E., McPhail K.L., Carter R.G. (2016). Enantioselective total synthesis of mandelalide A and isomandelalide A: Discovery of a cytotoxic ring-expanded isomer. J. Am. Chem. Soc..

[78] Nazari M., Serrill J.D., Sikorska J., Ye T., Ishmael J.E., McPhail K.L. (2016). Discovery of mandelalide E and determinants of cytotoxicity for the mandelalide series. Org. Lett..

[79] Nazari M., Serrill J.D., Wan X., Nguyen M.H., Anklin C., Gallegos D.A., Smith A.B., Ishmael J.E., McPhail K.L. (2017). New mandelalides expand a macrolide series of mitochondrial inhibitors. J. Med. Chem..

[80] Issac M., Aknin M., Gauvin-Bialecki A., Pond C.D., Barrows L.R., Kashman Y., Carmeli S. (2017). Mollecarbamates, Molleureas, and Molledihydroisoquinolone, o-Carboxyphenethylamide Metabolites of the Ascidian *Didemnum molle* Collected in Madagascar. J. Nat. Prod..

[81] Hayakawa I., Suzuki K., Okamura M., Funakubo S., Onozaki Y., Kawamura D., Ohyoshi T., Kigoshi H. (2017). Total Synthesis of Biselide E, a Marine Polyketide. Org. Lett..

[82] Menna M., Fattorusso E., Imperatore C. (2011). Alkaloids from marine ascidians. Molecules.

[83] Sakai R., Rinehart K.L., Guan Y., Wang A.H. (1992). Additional antitumor ecteinascidins from a Caribbean tunicate: Crystal structures and activities in vivo. Proc. Natl. Acad. Sci. USA.

[84] Le V.H., Inai M., Williams R.M., Kan T. (2015). Ecteinascidins. A review of the chemistry, biology and clinical utility of potent tetrahydroisoquinoline antitumor antibiotics. Nat. Prod. Rep..

[85] Gordon E.M., Sankhala K.K., Chawla N., Chawla S.P. (2016). Trabectedin for soft tissue sarcoma: Current status and future perspectives. Adv. Ther..

[86] D’Incalci M., Galmarini C.M. (2010). A review of trabectedin (ET-743): A unique mechanism of action. Mol. Cancer Ther..

[87] Jin S., Gorfajn B., Faircloth G., Scotto K.W. (2000). Ecteinascidin 743, a transcription-targeted chemotherapeutic that inhibits MDR1 activation. Proc. Natl. Acad. Sci. USA.

[88] D’Incalci M., Badri N., Galmarini C.M., Allavena P. (2014). Trabectedin, a drug acting on both cancer cells and the tumour microenvironment. Br. J. Cancer.

[89] Germano G., Frapolli R., Belgiovine C., Anselmo A., Pesce S., Liguori M., Erba E., Uboldi S., Zucchetti M., Pasqualini F. (2013). Role of macrophage targeting in the antitumor activity of trabectedin. Cancer Cell.

[90] Gomes N.G., Dasari R., Chandra S., Kiss R., Kornienko A. (2016). Marine invertebrate metabolites with anticancer activities: Solutions to the “supply problem”. Mar. Drugs.

[91] Leal J.F., Martínez-Díez M., García-Hernández V., Moneo V., Domingo A., Bueren-Calabuig J.A., Negri A., Gago F., Guillén-Navarro M.J., Avilés P. (2010). PM01183, a new DNA minor groove covalent binder with potent *in vitro* and *in vivo* anti-tumour activity. Brit. J. Pharmacol..

[92] Nuñez G.S., Robles C.M., Giraudon C., Martínez-Leal J.F., Compe E., Coin F., Aviles P., Galmarini C.M., Egly J.M. (2016). Lurbinectedin specifically triggers the degradation of phosphorylated RNA polymerase II and the formation of DNA breaks in cancer cells. Mol. Cancer Ther..

[93] Soares D.G., Machado M.S., Rocca C.J., Poindessous V., Ouaret D., Sarasin A., Galmarini C.M., Henriques J.A., Escargueil A.E., Larsen A.K. (2011). Trabectedin and its C subunit modified analogue PM01183 attenuate nucleotide excision repair and show activity toward platinum-resistant cells. Mol. Cancer Ther..

[94] Calvo E., Moreno V., Flynn M., Holgado E., Olmedo M.E., Lopez Criado M.P., Kahatt C., Lopez-Vilariño A., Siguero M., Fernandez-Teruel C. (2017). Antitumor activity of lurbinectedin (PM01183) and doxorubicin in relapsed small-cell lung cancer: Results from a phase I study. Ann. Oncol..

[95] Lima M., Bouzid H., Soares D.G., Selle F., Morel C., Galmarini C.M., Henriques J.A., Larsen A.K., Escargueil A.E. (2016). Dual inhibition of ATR and ATM potentiates the activity of trabectedin and lurbinectedin by perturbing the DNA damage response and homologous recombination repair. Oncotarget.

[96] Rath C.M., Janto B., Earl J., Ahmed A., Hu F.Z., Hiller L., Dahlgren M., Kreft R., Yu F., Wolff J.J. (2011). Meta-omic characterization of the marine invertebrate microbial consortium that produces the chemotherapeutic natural product ET-743. ACS Chem. Biol..

[97] Sherman D.H., Ehrlich G.D., Janto B., Williams R.M., Rath C.M. (2017). Allegheny-Singer Research Institute, Colorado State University Research Foundation and University of Michigan. Biosynthetic Pathway for Heterologous Expression of a Nonribosomal Peptide Synthetase Drug and Analogs. U.S. Patent.

[98] Sandjo L.P., Kuete V., Biavatti M.W. (2015). Pyridoacridine alkaloids of marine origin: NMR and MS spectral data, synthesis, biosynthesis and biological activity. Beilstein J. Org. Chem..

[99] Kijjoa A., Kim S.-K. (2015). Pyridoacridine Alkaloids from Marine Origin: Sources and Anticancer Activity. Handbook of Anticancer Drugs from Marine Origin.

[100] Hanahan D., Weinberg R.A. (2011). Hallmarks of cancer: The next generation. Cell.

[101] Ganesan K., Xu B. (2017). Telomerase Inhibitors from Natural Products and Their Anticancer Potential. Int. J. Mol. Sci..

[102] Mengual Gomez D.L., Armando R.G., Cerrudo C.S., Ghiringhelli P.D., Gomez D.E. (2016). Telomerase as a cancer target. Development of new molecules. Curr. Top. Med. Chem..

[103] Guittat L., De Cian A., Rosu F., Gabelica V., De Pauw E., Delfourne E., Mergny J.L. (2005). Ascididemin and meridine stabilise G-quadruplexes and inhibit telomerase in vitro. Biochim. Biophys. Acta (BBA)-Gen. Subj..

[104] Delfourne E., Darro F., Bontemps-Subielos N., Decaestecker C., Bastide J., Frydman A., Kiss R. (2001). Synthesis and characterization of the antitumor activities of analogues of meridine, a marine pyridoacridine alkaloid. J. Med. Chem..

[105] Sharma V., Sharma P.C., Kumar V. (2016). *In Silico* Molecular Docking Analysis of Natural Pyridoacridines as Anticancer Agents. Adv. Chem..

[106] Plodek A., Bracher F. (2016). New Perspectives in the Chemistry of Marine Pyridoacridine Alkaloids. Mar. Drugs.

[107] Rinehart K.L., Kobayashi J., Harbour G.C., Hughes R.G., Mizsak S.A., Scahill T.A. (1984). Eudistomins C, E, K, and L, potent antiviral compounds containing a novel oxathiazepine ring from the Caribbean tunicate *Eudistoma olivaceum*. J. Amer. Chem. Soc..

[108] Giulietti J.M., Tate P.M., Cai A., Cho B., Mulcahy S.P. (2016). DNA-binding studies of the natural β-carboline eudistomin U. Bioorg. Med. Chem. Lett..

[109] Ota Y., Chinen T., Yoshida K., Kudo S., Nagumo Y., Shiwa Y., Yamada R., Umihara H., Iwasaki K., Masumoto H. (2016). Eudistomin C, an antitumor and antiviral natural product, targets 40S ribosome and inhibits protein translation. ChemBioChem.

[110] Kumar S., Singh A., Kumar K., Kumar V. (2017). Recent insights into synthetic β-carbolines with anti-cancer activities. Eur. J. Med. Chem..

[111] Laine A.E., Lood C., Koskinen A.M. (2014). Pharmacological importance of optically active tetrahydro-β-carbolines and synthetic approaches to create the C1 stereocenter. Molecules.

[112] Panarese J.D., Waters S.P. (2010). Room-temperature aromatization of tetrahydro-β-carbolines by 2-iodoxybenzoic acid: Utility in a total synthesis of Eudistomin U. Org. Lett..

[113] Fan H., Peng J., Hamann M.T., Hu J.F. (2008). Lamellarins and related pyrrole-derived alkaloids from marine organisms. Chem. Rev..

[114] Marco E., Laine W., Tardy C., Lansiaux A., Iwao M., Ishibashi F., Bailly C., Gago F. (2005). Molecular Determinants of Topoisomerase I Poisoning by Lamellarins: Comparison with Camptothecin and Structure–Activity Relationships. J. Med. Chem..

[115] Bailly C. (2015). Anticancer properties of lamellarins. Mar. Drugs.

[116] Khiati S., Seol Y., Agama K., Dalla Rosa I., Agrawal S., Fesen K., Zhang H., Neuman K.C., Pommier Y. (2014). Poisoning of mitochondrial topoisomerase I by lamellarin D. Mol. Pharmacol..

[117] Ballot C., Martoriati A., Jendoubi M., Buche S., Formstecher P., Mortier L., Kluza J., Marchetti P. (2014). Another facet to the anticancer response to lamellarin D: Induction of cellular senescence through inhibition of topoisomerase I and intracellular ROS production. Mar. Drugs.

[118] Long S., Sousa E., Kijjoa A., Pinto M.M. (2016). Marine natural products as models to circumvent multidrug resistance. Molecules.

[119] Huang X., Kumar P., Anreddy N., Xiao X., Yang D.H., Chen Z.S., Kim S.-K. (2015). P-gp Inhibitory Activity from Marine Sponges, Tunicates and Algae. Handbook of Anticancer Drugs from Marine Origin.

[120] Plisson F., Huang X.C., Zhang H., Khalil Z., Capon R.J. (2012). Lamellarins as Inhibitors of P-Glycoprotein-Mediated Multidrug Resistance in a Human Colon Cancer Cell Line. Chem. Asian J..

[121] Imbri D., Tauber J., Opatz T. (2014). Synthetic approaches to the lamellarins—A comprehensive review. Mar. Drugs.

[122] Lade D.M., Pawar A.B., Mainkar P.S., Chandrasekhar S. (2017). Total Synthesis of Lamellarin D Trimethyl Ether, Lamellarin D, and Lamellarin H. J. Org. Chem..

[123] Manjappa K.B., Lin J.M., Yang D.Y. (2017). Construction of Pentacyclic Lamellarin Skeleton via Grob Reaction: Application to Total Synthesis of Lamellarins H and D. J. Org. Chem..

[124] Yang C., Wong I.L., Peng K., Liu Z., Wang P., Jiang T., Jiang T., Chow L.M., Wan S.B. (2017). Extending the structure–activity relationship study of marine natural ningalin B analogues as P-glycoprotein inhibitors. Eur. J. Med. Chem..

[125] Plisson F., Conte M., Khalil Z., Huang X.C., Piggott A.M., Capon R.J. (2012). Kinase inhibitor scaffolds against neurodegenerative diseases from a Southern Australian ascidian, *Didemnum* sp. ChemMedChem.

[126] Kim J.Y., Kim D.H., Jeon T.H., Kim W.H., Cho C.G. (2017). Total Syntheses of Ningalins D and G. Org. Lett..

[127] Cherigo L., Lopez D., Martinez-Luis S. (2015). Marine natural products as breast cancer resistance protein inhibitors. Mar. Drugs.

[128] Takada K., Imamura N., Gustafson K.R., Henrich C.J. (2010). Synthesis and structure–activity relationship of botryllamides that block the ABCG2 multidrug transporter. Bioorg. Med. Chem. Lett..

[129] Yadav R.R., Sharma S., Joshi P., Wani A., Vishwakarma R.A., Kumar A., Bharate S.B. (2015). Meridianin derivatives as potent Dyrk1A inhibitors and neuroprotective agents. Bioorg. Med. Chem. Lett..

[130] Bharate S.B., Yadav R.R., Battula S., Vishwakarma A.R. (2012). Meridianins: Marine-derived potent kinase inhibitors. Mini Rev. Med. Chem..

[131] More K.N., Jang H.W., Hong V.S., Lee J. (2014). Pim kinase inhibitory and antiproliferative activity of a novel series of meridianin C derivatives. Bioorg. Med. Chem. Lett..

[132] Park Y.K., Lee T.Y., Choi J.S., Hong V.S., Lee J., Park J.W., Jang B.C. (2014). Inhibition of adipogenesis and leptin production in 3T3-L1 adipocytes by a derivative of meridianin C. Biochem. Biophys. Res. Commun..

[133] Núñez-Pons L., Nieto R.M., Avila C., Jiménez C., Rodríguez J. (2015). Mass spectrometry detection of minor new meridianins from the antarctic colonial ascidians *Aplidium falklandicum* and *Aplidium meridianum*. J. Mass Spectrom..

[134] Sandtorv A.H., Atta-ur-Rahman (2017). Chemical Synthesis of Meridianins and Related Derivatives. Studies in Natural Products Chemistry.

[135] Jarry M., Lecointre C., Malleval C., Desrues L., Schouft M.T., Lejoncour V., Liger F., Lyvinec G., Joseph B., Loaëc N. (2014). Impact of meriolins, a new class of cyclin-dependent kinase inhibitors, on malignant glioma proliferation and neo-angiogenesis. Neuro-Oncol..

[136] Liberio M.S., Sadowski M.C., Davis R.A., Rockstroh A., Vasireddy R., Lehman M.L., Nelson C.C. (2015). The ascidian natural product eusynstyelamide B is a novel topoisomerase II poison that induces DNA damage and growth arrest in prostate and breast cancer cells. Oncotarget.

[137] Chan S.T., Patel P.R., Ransom T.R., Henrich C.J., McKee T.C., Goey A.K., Cook K.M., Figg W.D., McMahon J.B., Schnermann M.J. (2015). Structural Elucidation and Synthesis of Eudistidine A: An Unusual Polycyclic Marine Alkaloid that Blocks Interaction of the Protein Binding Domains of p300 and HIF-1α. J. Am. Chem. Soc..

[138] Chan S.T., Nani R.R., Schauer E.A., Martin G.E., Williamson R.T., Saurí J., Buevich A.V., Schafer W.A., Joyce L.A., Goey A.K. (2016). Characterization and Synthesis of Eudistidine C, a Bioactive Marine Alkaloid with an Intriguing Molecular Scaffold. J. Org. Chem..

[139] Tatsuta T., Hosono M., Rotinsulu H., Wewengkang D.S., Sumilat D.A., Namikoshi M., Yamazaki H. (2017). Lissoclibadin 1, a Polysulfur Aromatic Alkaloid from the Indonesian Ascidian *Lissoclinum cf. badium*, induces Caspase-Dependent Apoptosis in Human Colon Cancer Cells and Suppresses Tumor Growth in Nude Mice. J. Nat. Prod..

[140] Oda T., Fujiwara T., Liu H., Ukai K., Mangindaan R.E., Mochizuki M., Namikoshi M. (2006). Effects of Lissoclibadins and Lissoclinotoxins, Isolated from a Tropical Ascidian Lissoclinum cf. badium, on IL-8 production in a PMA-stimulated Promyelocytic Leukemia Cell Line. Mar. Drugs.

[141] Fukuzawa S., Matsunaga S., Fusetani N. (1997). Isolation of 13 New Ritterazines from the Tunicate *Ritterella tokioka* and Chemical Transformation of Ritterazine B1. J. Org. Chem..

[142] Lee S., LaCour T.G., Fuchs P.L. (2009). Chemistry of trisdecacyclic pyrazine antineoplastics: The cephalostatins and ritterazines. Chem. Rev..

[143] Ambrose A.J., Santos E.A., Jimenez P.C., Rocha D.D., Wilke D.V., Beuzer P., Axelrod J., Kanduluru A.K., Fuchs P.L., Cang H. (2017). Ritterostatin GN1N, a Cephalostatin–Ritterazine Bis-steroidal Pyrazine Hybrid, Selectively Targets GRP78. ChemBioChem.

[144] Tahtamouni L.H., Nawasreh M.M., Al-Mazaydeh Z.A., Al-Khateeb R.A., Abdellatif R.N., Bawadi R.M., Bamburg J.R., Yasin S.R. (2018). Cephalostatin 1 analogues activate apoptosis via the endoplasmic reticulum stress signaling pathway. Eur. J. Pharmacol..

[145] Shi Y., Jia L., Xiao Q., Lan Q., Tang X., Wang D., Li M., Ji Y., Zhou T., Tian W. (2011). A practical synthesis of cephalostatin 1. Chem. Asian J..

[146] Shi Y., Jiang X.L., Tian W.S. (2017). Synthesis of 12,12′-azo-13,13′-diepi-Ritterazine N. J. Org. Chem..

[147] Piccialli V. (2014). Ruthenium tetroxide and perruthenate chemistry. Recent advances and related transformations mediated by other transition metal oxo-species. Molecules.

[148] Tran T.D., Pham N.B., Ekins M., Hooper J.N., Quinn R.J. (2015). Isolation and total synthesis of stolonines A–C, unique taurine amides from the Australian marine tunicate *Cnemidocarpa stolonifera*. Mar. Drugs.

[149] Won T.H., Kim C.K., Lee S.H., Rho B.J., Lee S.K., Oh D.C., Oh K.B., Shin J. (2015). Amino Acid-Derived Metabolites from the Ascidian *Aplidium* sp. Mar. Drugs.

[150] Biard J.F., Malochet-Grivois C., Roussakis C., Cotelle P., Hénichart J.P., Débitus C., Verbist J.F. (1994). Lissoclimides, cytotoxic diterpenes from *Lissoclinum voeltzkowi* Michaelsen. Nat. Prod. Lett..

[151] Könst Z.A., Szklarski A.R., Pellegrino S., Michalak S.E., Meyer M., Zanette C., Cencic R., Nam S., Voora V.K., Horne D.A. (2017). Synthesis facilitates an understanding of the structural basis for translation inhibition by the lissoclimides. Nat. Chem..

[152] Gandhi V., Plunkett W., Cortes J.E. (2014). Omacetaxine: A protein translation inhibitor for treatment of chronic myelogenous leukemia. Clin. Cancer Res..

[153] Quinn R.K., Könst Z.A., Michalak S.E., Schmidt Y., Szklarski A.R., Flores A.R., Nam S., Horne D.A., Vanderwal C.D., Alexanian E.J. (2016). Site-selective aliphatic C–H chlorination using N-chloroamides enables a synthesis of chlorolissoclimide. J. Am. Chem. Soc..

[154] Imperatore C., Cimino P., Cebrián-Torrejón G., Persico M., Aiello A., Senese M., Fattorusso C., Menna M., Doménech-Carbó A. (2017). Insight into the Mechanism of Action of Marine Cytotoxic Thiazinoquinones. Mar. Drugs.

[155] Aiello A., Fattorusso E., Luciano P., Macho A., Menna M., Muñoz E. (2005). Antitumor effects of two novel naturally occurring terpene quinones isolated from the Mediterranean ascidian *Aplidium conicum*. J. Med. Chem..

[156] Harper J.L., Khalil I.M., Shaw L., Bourguet-Kondracki M.L., Dubois J., Valentin A., Barker D., Copp B.R. (2015). Structure–activity relationships of the bioactive thiazinoquinone marine natural products thiaplidiaquinones A and B. Mar. Drugs.

[157] Cadelis M.M., Bourguet-Kondracki M.L., Dubois J., Kaiser M., Brunel J.M., Barker D., Copp B.R. (2017). Structure–activity relationship studies on thiaplidiaquinones A and B as novel inhibitors of Plasmodium falciparum and farnesyltransferase. Bioorg. Med. Chem..

[158] Possner S.T., Schroeder F.C., Rapp H.T., Sinnwell V., Franke S., Francke W. (2017). 3, 7-Isoquinoline quinones from the ascidian tunicate *Ascidia virginea*. Z. Naturforsch. C..

[159] Lien W.C., Chen T.Y., Sheu S.Y., Lin T.C., Kang F.C., Yu C.H., Kuan T.S., Huang B.M., Wang C.Y. (2017). 7-hydroxy-staurosporine, UCN-01, induces DNA damage response and autophagy in human osteosarcoma U2-OS cells. J. Cell. Biochem..

[160] Jiang X., Zhao B., Britton R., Lim L.Y., Leong D., Sanghera J.S., Zhou B.B., Piers E., Andersen R.J., Roberge M. (2004). Inhibition of Chk1 by the G2 DNA damage checkpoint inhibitor isogranulatimide. Mol. Cancer Ther..

[161] Lavrard H., Rodriguez F., Delfourne E. (2014). Design of granulatimide and isogranulatimide analogues as potential Chk1 inhibitors: Study of amino-platforms for their synthesis. Bioorg. Med. Chem..

[162] Lavrard H., Salvetti B., Mathieu V., Rodriguez F., Kiss R., Delfourne E. (2015). Synthesis and in vitro antiproliferative activity of amido and amino analogues of the marine alkaloid isogranulatimide. ChemMedChem.

[163] Deslandes S., Chassaing S., Delfourne E. (2009). Marine pyrrolocarbazoles and analogues: Synthesis and kinase inhibition. Mar. Drugs.

[164] Loaëc N., Attanasio E., Villiers B., Durieu E., Tahtouh T., Cam M., Davis R.A., Alencar A., Roué M., Bourguet-Kondracki M.L. (2017). Marine-Derived 2-Aminoimidazolone Alkaloids. Leucettamine B-Related Polyandrocarpamines Inhibit Mammalian and Protozoan DYRK & CLK Kinases. Mar. Drugs.

[165] Oddo S., LaFerla F.M. (2006). The role of nicotinic acetylcholine receptors in Alzheimer’s disease. J. Physiol.-Paris.

[166] Llorach-Pares L., Nonell-Canals A., Sanchez-Martinez M., Avila C. (2017). Computer-Aided Drug Design Applied to Marine Drug Discovery: Meridianins as Alzheimer’s Disease Therapeutic Agents. Mar. Drugs.

[167] Dev K., Maurya R., Brahmachari G. (2017). Marine-Derived Anti-Alzheimer’s Agents of Promise. Neuroprotective Natural Products: Clinical Aspects and Mode of Action.

[168] Tadesse M., Svenson J., Sepčić K., Trembleau L., Engqvist M., Andersen J.H., Jaspars M., Stensvåg K., Haug T. (2014). Isolation and synthesis of pulmonarins A and B, acetylcholinesterase inhibitors from the colonial ascidian *Synoicum pulmonaria*. J. Nat. Prod..

[169] Tadokoro Y., Nishikawa T., Ichimori T., Matsunaga S., Fujita M.J., Sakai R. (2017). N-Methyl-β-carbolinium Salts and an N-Methylated 8-Oxoisoguanine as Acetylcholinesterase Inhibitors from a Solitary Ascidian, *Cnemidocarpa irene*. ACS Omega.

[170] Kudryavtsev D., Makarieva T., Utkina N., Santalova E., Kryukova E., Methfessel C., Tsetlin V., Stonik V., Kasheverov I. (2014). Marine natural products acting on the acetylcholine-binding protein and nicotinic receptors: From computer modeling to binding studies and electrophysiology. Mar. Drugs.

[171] Tsuneki H., You Y., Toyooka N., Sasaoka T., Nemoto H., Dani J.A., Kimura I. (2005). Marine alkaloids (−)-pictamine and (−)-lepadin B block neuronal nicotinic acetylcholine receptors. Biol. Pharm. Bull..

[172] Li X., Hu L., Jia J., Gu H., Jia Y., Chen X. (2017). A Stereoselective Approach toward (−)-Lepadins A–C. Org. Lett..

[173] Lazo J.S., McQueeney K.E., Burnett J.C., Wipf P., Sharlow E.R. (2018). Small molecule targeting of PTPs in cancer. Int. J. Biochem. Cell Biol..

[174] Zhou Y., Zhang W., Liu X., Yu H., Lu X., Jiao B. (2017). Inhibitors of Protein Tyrosine Phosphatase 1B from Marine Natural Products. Chem. Biodivers..

[175] Yamazaki H., Nakayama W., Takahashi O., Kirikoshi R., Izumikawa Y., Iwasaki K., Toraiwa K., Ukai K., Rotinsulu H., Wewengkang D.S. (2015). Verruculides A and B, two new protein tyrosine phosphatase 1B inhibitors from an Indonesian ascidian-derived *Penicillium verruculosum*. Bioorg. Med. Chem. Lett..

[176] Sumilat D.A., Yamazaki H., Endo K., Rotinsulu H., Wewengkang D.S., Ukai K., Namikoshi M. (2017). A new biphenyl ether derivative produced by Indonesian ascidian-derived *Penicillium albobiverticillium*. J. Nat. Med..

[177] Imperatore C., Luciano P., Aiello A., Vitalone R., Irace C., Santamaria R., Li J., Guo Y.W., Menna M. (2016). Structure and Configuration of Phosphoeleganin, a Protein Tyrosine Phosphatase 1B Inhibitor from the Mediterranean Ascidian *Sidnyum elegans*. J. Nat. Prod..

[178] Luciano P., Imperatore C., Senese M., Aiello A., Casertano M., Guo Y.W., Menna M. (2017). Assignment of the Absolute Configuration of Phosphoeleganin via Synthesis of Model Compounds. J. Nat. Prod..

[179] Miller J.H., Field J.J., Kanakkanthara A., Owen J.G., Singh A.J., Northcote P.T. (2018). Marine Invertebrate Natural Products that Target Microtubules. J. Nat. Prod..

[180] Cruz-Monserrate Z., Vervoort H.C., Bai R., Newman D.J., Howell S.B., Los G., Mullaney J.T., Williams M.D., Pettit G.R., Fenical W. (2003). Diazonamide A and a synthetic structural analog: Disruptive effects on mitosis and cellular microtubules and analysis of their interactions with tubulin. Mol. Pharm..

[181] Wieczorek M., Tcherkezian J., Bernier C., Prota A.E., Chaaban S., Rolland Y., Godbout C., Hancock M.A., Arezzo J.C., Ocal O. (2016). The synthetic diazonamide DZ-2384 has distinct effects on microtubule curvature and dynamics without neurotoxicity. Sci. Transl. Med..

[182] David N., Pasceri R., Kitson R.R., Pradal A., Moody C.J. (2016). Formal Total Synthesis of Diazonamide A by Indole Oxidative Rearrangement. Chem. Eur. J..

[183] Griffiths G., Garrone B., Deacon E., Owen P., Pongracz J., Mead G., Bradwell A., Watters D., Lord J. (1996). The Polyether Bistratene A Activates Protein Kinase C–δ and Induces Growth Arrest in HL60 Cells. Biochem. Biophys. Res. Commun..

[184] Statsuk A.V., Bai R., Baryza J.L., Verma V.A., Hamel E., Wender P.A., Kozmin S.A. (2005). Actin is the primary cellular receptor of bistramide A. Nat. Chem. Biol..

[185] Rizvi S.A., Tereshko V., Kossiakoff A.A., Kozmin S.A. (2006). Structure of Bistramide A—Actin Complex at a 1.35 Å Resolution. J. Amer. Chem. Soc..

[186] Rizvi S.A., Courson D.S., Keller V.A., Rock R.S., Kozmin S.A. (2008). The dual mode of action of bistramide A entails severing of filamentous actin and covalent protein modification. Proc. Natl. Acad. Sci. USA.

[187] Rizvi S.A., Liu S., Chen Z., Skau C., Pytynia M., Kovar D.R., Chmura S.J., Kozmin S.A. (2010). Rationally simplified bistramide analog reversibly targets actin polymerization and inhibits cancer progression in vitro and in vivo. J. Am. Chem. Soc..

[188] Sauviat M.P., Gouiffes-Barbin D., Ecault E., Verbist J.F. (1992). Blockade of sodium channels by bistramide A in voltage-clamped frog skeletal muscle fibres. Biochim. Biophys. Acta (BBA)-Biomembranes.

[189] Wrona I.E., Lowe J.T., Turbyville T.J., Johnson T.R., Beignet J., Beutler J.A., Panek J.S. (2009). Synthesis of a 35-member stereoisomer library of bistramide A: Evaluation of effects on actin state, cell cycle and tumor cell growth. J. Org. Chem..

[190] Herkommer D., Dreisigacker S., Sergeev G., Sasse F., Gohlke H., Menche D. (2015). Design, synthesis, and biological evaluation of simplified side chain hybrids of the potent actin binding polyketides rhizopodin and bistramide. ChemMedChem.

[191] Zhang F.M., Zhang S.Y., Tu Y.Q. (2018). Recent progress in the isolation, bioactivity, biosynthesis, and total synthesis of natural spiroketals. Nat. Prod. Rep..

[192] Jaffarali H.A., Akram S., Arshan K.M. (2017). DNA barcoding of a colonial ascidian, *Lissoclinum fragile* (Van Name, 1902). Mitochondrial DNA Part A.

[193] Jaffarali H.A., Akram S., Arshan K.M. (2018). Identification of four Indian ascidians based on COI gene sequences. Mitochondrial DNA Part A.

[194] Eastman A. (2017). Improving anticancer drug development begins with cell culture: Misinformation perpetrated by the misuse of cytotoxicity assays. Oncotarget.

[195] Newman D.J. (2016). Developing natural product drugs: Supply problems and how they have been overcome. Pharmacol. Ther..

[196] Schofield M.M., Jain S., Porat D., Dick G.J., Sherman D.H. (2015). Identification and analysis of the bacterial endosymbiont specialized for production of the chemotherapeutic natural product ET-743. Environ. Microbiol..

[197] Sardar D., Tianero M.D., Schmidt E.W. (2016). Directing biosynthesis: Practical supply of natural and unnatural cyanobactins. Methods in Enzymology.

[198] Zhang G., Li J., Zhu T., Gu Q., Li D. (2016). Advanced tools in marine natural drug discovery. Curr. Opin. Biotechnol..

